# Histone Citrullination Mediates a Protective Role in Endothelium and Modulates Inflammation

**DOI:** 10.3390/cells11244070

**Published:** 2022-12-15

**Authors:** Rebeca Osca-Verdegal, Jesús Beltrán-García, Ana B. Paes, Elena Nacher-Sendra, Susana Novella, Carlos Hermenegildo, Nieves Carbonell, José Luis García-Giménez, Federico V. Pallardó

**Affiliations:** 1 Centro de Investigación Biomédica en Red de Enfermedades Raras (CIBERER), Instituto de Salud Carlos III, 46010 Valencia, Spain; 2 Departamento de Fisiología, Facultad de Medicina y Odontología, Universitat de València, 46010 València, Spain; 3 Systems Neurobiology Laboratory, Salk Institute for Biological Studies, La Jolla, CA 92037, USA; 4 Department of Medicine, Division of Regenerative Medicine, University of California, La Jolla, CA 92037, USA; 5 Instituto de Investigación Sanitaria INCLIVA, 46010 Valencia, Spain; 6 Hospital Clínic Universitari de València, 46010 Valencia, Spain; 7 EpiDisease S.L. (Spin-Off CIBER-ISCIII), Parc Científic de la Universitat de València, 46980 Paterna, Spain

**Keywords:** citrullination, histones, NETosis, sepsis, septic shock, progression, biomarker

## Abstract

NETosis is a key host immune process against a pathogenic infection during innate immune activation, consisting of a neutrophil “explosion” and, consequently, NET formation, containing mainly DNA, histones, and other nuclear proteins. During sepsis, an exacerbated immune host response to an infection occurs, activating the innate immunity and NETosis events, which requires histone H3 citrullination. Our group compared the circulating histone levels with those citrullinated H3 levels in plasma samples of septic patients. In addition, we demonstrated that citrullinated histones were less cytotoxic for endothelial cells than histones without this post-translational modification. Citrullinated histones did not affect cell viability and did not activate oxidative stress. Nevertheless, citrullinated histones induced an inflammatory response, as well as regulatory endothelial mechanisms. Furthermore, septic patients showed elevated levels of circulating citrullinated histone H3, indicating that the histone citrullination is produced during the first stages of sepsis, probably due to the NETosis process.

## 1. Introduction

Sepsis is defined as a dysregulated host response that damages the host’s own tissues in response to bacterial, fungal, or viral infections. Likewise, septic shock (SS) is a subset of sepsis in which circulatory, cellular, and metabolic abnormalities are associated with a higher risk of mortality [1]. Sepsis is one of the most common global illnesses and is the leading cause of death in intensive care units (ICUs) worldwide [2]. Specifically, 48 million cases of sepsis are estimated every year, producing about 11 million deaths, even more than myocardial infarction [3], and the number of cases is increasing every year.

During the host defense process against invading pathogens, one of the first events activated by immunity is ETosis. This is an active innate immune process via which neutrophils and macrophages release their content, including microbiocidal proteins, nucleic acids, and nuclear proteins, mainly histones. The most characterized mechanism is NETosis, mediated by neutrophils. NETosis releases an extracellular fibril matrix known as NETs (neutrophil extracellular traps), which bind to pathogens and neutralize them, thus helping impair infections [4,5,6]. The formation of NETs is a very effective immune strategy to eliminate pathogens; however, if they are not properly regulated, NETs can propagate inflammation and microvascular thrombosis contributing to the pathophysiology of sepsis [7]. Because NETs are released into the bloodstream, the endothelium is the first tissue to be exposed to NETs; hence, endothelial cells are the first cell type in contact with them, as well as their components such as histones.

Histones are nuclear proteins that act as chromosomal organizers to pack DNA and regulate gene expression through different epigenetic mechanisms, mainly PTMs (post-translational modifications). Under physiological conditions, histones are found in the nucleus, packaging the DNA, and they are, therefore, present at very low concentrations in the extracellular space. However, when released outside of the cell, extracellular histones can act as damage-associated molecular patterns (DAMPs), activating several signaling pathways [8,9]. These molecular events can originate a devastating cellular death process affecting several cell and tissue functions, subsequently enhancing inflammation and contributing to the poor progression of critically ill patients [10]. In this regard, different histone PTMs have been described to modulate the potential of histones to act as DAMPs, guiding an increase or decrease in the cytotoxic capacity of extracellular histones [11]. Among them, histone citrullination stands out in sepsis because H3 histone citrullination is an initial and critical step for NETosis activation, playing a central role in modulating the NETosis [12,13]. The citrullination of histone H3 is catalyzed by peptidylarginine deiminases (PAD), with PAD4 being the most important isoform, although it has been shown that PAD2 can also assist in this process [14,15]. Furthermore, it has been demonstrated that PAD4 is also able to citrullinate histones H4 and H2A [16,17,18,19], as well as H1 [20]. Therefore, since H3 citrullination is the first and critical event of NETosis, it is expected that a large proportion of H3 histones, as well as several other histones (mainly H4 and H2A) are released into the bloodstream in the early stages of sepsis in their citrullinated form.

Regarding the different tissues affected during sepsis, the endothelium is one of the most affected, since it is the first one to face the different stimuli such as the invading pathogens, as well as the immune host response, including the exposure to extracellular histones and other nuclear proteins, NETs, and other DAMPs, ultimately affecting its normal function [21]. Specifically, endothelial cells amplify the immune response and activate the coagulation system [22], by releasing enzymes that control blood clotting and leukocyte and platelet adhesion [23,24]. Furthermore, the endothelium provides a linkage between local and systemic immune responses, since it is both a source of and a target for inflammation [25]. Likewise, the endothelial function has an impact on vascular homeostasis and activation of processes such as thrombosis, inflammation, and vascular remodeling [26,27,28,29], processes that all take place during sepsis pathophysiology. Moreover, endothelial cells release mediators that control vascular relaxation and contraction, as well as enzymes that control blood clotting, immune function, leukocyte, and platelet adhesion [30]. In this regard, endothelial damage plays a central role in the progression to organ failure during sepsis, and it is a major contributor to sepsis mortality [31,32,33]. Therefore, counteracting endothelial dysfunction has been demonstrated to improve sepsis outcomes [34,35,36].

Nonetheless, despite the important role of extracellular histones in sepsis that has already been described [37], how histone PTMs and main citrullination affect the endothelium has not yet been elucidated. In this work, we analyzed the role of citrullinated histones, analyzing their presence in septic patients’ bloodstreams, and we explored the role that they are playing during sepsis development and progression via statistical correlations with different clinical parameters. Additionally, we characterize the different molecular mechanisms describing how citrullinated histones on human endothelial cells and how citrullination can regulate the endothelial responses mediated by histones, as well as the loss of endothelial homeostasis, thus postulating histone citrullination as a potential target to treat sepsis.

## 2. Materials and Methods

### 2.1. Patient Selection

Eighty-eight plasma samples collected in the time course of the sepsis process from 26 patients were analyzed: 11 sepsis survivors (36 samples), 12 SS survivors (43 samples), and three SS non-survivors (nine samples). All septic patients met the Sepsis-3 Consensus criteria [1]. Samples were collected during the first 24 h of patient admission in the ICU (first sample), after 3 days (second sample), after 5 days (third sample), and pre-discharge or pre-exitus (before death) (last sample). The SS survivor group is a group of septic patients with a severe phenotype, although they survived SS.

Practitioners in the ICU at the Hospital Clínico Universitario of Valencia (HCUV, Spain) established Acute Physiology and Chronic Health Evaluation II (APACHE II) and Sequential Organ Failure Assessment (SOFA), as well as different clinical, microbiological, hemodynamic, and biochemical determinations, in each patient. The patients’ plasma samples were obtained from INCLIVA’s Biobank. All experimental protocols and methods were performed after obtaining approval from the HCUV’s Biomedical Research Ethics Committee. All participants signed the written informed consent form. The methods were performed in line with relevant international guidelines and regulations.

Septic patients were clinically defined because they met at least two of the following criteria: temperature greater than 38 °C or less than 36 °C, systolic blood pressure ≤100 mmHg, and leukocyte count >12,000/mm^3^ or <4000/mm^3^, while also differentiating SS by lactate levels ≥2 mmol/L (>18 mg/dL) in the absence of hypovolemia, and requiring vasopressor treatment to maintain blood pressure at 65 mmHg [4].

### 2.2. Analysis of Histone Levels in Plasma Samples

Plasma samples were centrifuged for delipidation for 15 min at 15,000× *g*. For each sample, 2 μL was diluted in 8 μL of H_2_O. Then, 2 μL of each 1/5 diluted plasma (25 μg) sample was mixed in 18 μL of 50 mM ammonium bicarbonate with 25 fmol of Heavy Mix peptides in a final volume of 20 μL. The cysteine residues were reduced using 2 mM DTT (DL-dithiothreitol) in 50 mM ammonium bicarbonate for 3 min at 300 W in a microwave. Sulfhydryl groups were alkylated with 5 mM iodoacetamide (IAM) using 50 mM ammonium bicarbonate in the dark for 20 min at room temperature. The reaction was stopped with trifluoroacetic acid (TFA) at a final concentration of 1%.

To perform mass spectrometry analysis, we used a 5500QTRAP hybrid triple-quadrupole/linear ion trap mass spectrometer (ABSIEX), equipped with a Micro M3 MicroLC chromatographic system. For that, we injected 10 μL of tryptic digestion (about 9 μg of protein and 25 fmol of each spike-in peptide) into a Trap column (10 × 0.3 mm Trap Cartridge Chromxp C18CL 5 µm, ABSCIEX) and separated using an analytical column (ChromXP C18, 120 A, 3 μm, 150 × 0.3 mm). Elution was carried out with a linear gradient of 0% to 35% B in A for 30 min (A: 0.1% FA; B: ACN, 0.1% FA) at a flow rate of 5 μL/min.

The 5500 QTRAP was operated in MRM mode. MRM data were acquired with a spray voltage of 5500 V in the positive mode, curtain gas at 25 psi, ion source gas at 25 psi, entrance potential (EP) of10, and exit potential (EXP) of 16. The declustering potential (DP) and collision energy (CE) were optimized during each transition. Relative quantification of each histone was performed using the area ratios (light/heavy) for all transitions, calculating them using Skyline software (MacCoss lab. Skyline version 4.2.1.19058; Mineapolis, MN, 2012), and light concentration was estimated as fmol/μL of initial serum and eventually converted to ng/mL of protein.

For the absolute quantification of circulating histones in plasma samples, we used the following peptides isotopically labeled: SpikeTides™ TQL LLLPGELAK and STELLIR, as shown in Table 1. SpikeTides™ TQL consisted of heavy-isotopically labeled proteotypic peptides that terminated with a C-terminal heavy Lys: 13C6, 15N2; Arg: 13C6, 15N4 (JPT Peptide Technologies, Berlin, Germany) for MRM-MS quantification.

### 2.3. Analysis of H3 Citrullination

Circulating H3 citrullination levels from plasma samples were measured using an EpiQuik Histone H3 Citrullination ELISA Kit (EpiGentek, Farmingdale, NY, USA), following the manufacturer’s specifications.

### 2.4. Cell Culture and Experimental Design

HUVECs (human umbilical vein endothelial cells) were isolated from umbilical cords of healthy neonates, following the principles outlined in the Declaration of Helsinki, approved by the Ethical Committee of Clinical Research of the Hospital Clínico Universitario of Valencia (HCUV, Spain).

HUVECs were isolated from human umbilical veins treated with 1% collagenase (Life Technologies, Carlsbad, CA, USA) and were cultured in specific growth medium 199 (M199) with Earle’s Balanced Salt Solution and L-glutamine (Lonza, Verviers, Belgium), supplemented with 20% fetal bovine serum (FBS) (Invitrogen, San Diego, CA, USA), 1% penicillin/amphotericin (Invitrogen, San Diego, CA, USA), and growth factors (Sigma-Aldrich, Tokyo, Japan). Cells used in this work had 3–6 passages and were incubated at 37 °C in a Thermo Scientific incubator, Heracell 150i CO_2_ incubator (Thermo Scientific, New York, NY, USA) in a humidified atmosphere (5% CO_2_).

### 2.5. Histone Extraction, Purification, and In Vitro Citrullination

HeLa cells were used for obtaining human histones. Those cells were cultured with Iscove’s Dulbecco’s modified Eagle medium (DMEM) with high glucose (Invitrogen, San Diego, CA, USA), supplemented with 10% FBS (Invitrogen, San Diego, CA, USA) and 1% penicillin/streptomycin (Invitrogen, San Diego, CA, USA).

Histones were purified using the protocol described by Shechter et al., (2007) [38], with some modifications. Briefly, HeLa cells were resuspended in Hypotonic Lysis Buffer (10 mM Tris-HCl pH = 8, 1 mM KCl, 1.5 mM MgCl_2_) with 10 μL/mL of orthovanadate, and 2 μL/mL of protease inhibitor (Fisher Scientific, Hampton, SC, USA). Afterward, cells were kept in rotation for 30 min at 4 °C and then were centrifuged for 10 min at 4 °C at 10,000 rpm. Finally, the pellet was resuspended in H_2_SO_4_ (0.4 N) and was kept in rotation overnight at 4 °C. The next day, after centrifugation, the supernatant was kept and mixed with tricarboxylic acid (100%) and incubated on ice for 30 min. Then, the sample was centrifuged, and the pellet was washed with acetone. Finally, after the final centrifugation, the pellet was resuspended in H_2_O.

After HeLa histone extraction, citrullination of histones was induced using PAD Cocktail Active (SignalChem, Richmond, BC, Canadá) following the manufacturer’s instructions. Different ratios of histone/PAD were tested (histone/PAD Cocktail Active- 1:1; 1:0,5; 1:0,25; 1:0) to identify the strongest citrullination induction, using H3 histone citrullination as an indicator of total citrullination levels (Supplementary Figure S1). Finally, a ratio 1:1 of histones and PAD Cocktail Active was selected as the working concentration to induce histone citrullination.

### 2.6. Cell Viability and Cell Death Determination

A preliminary study of viability was conducted using the MTT (3-(4,5-dimethylthiazol-2-yl)-2,5-diphenyl tetrazolium bromide) method. For this assay, 5 mg of MTT (Sigma-Aldrich, Tokyo, Japan) salt was diluted in 1 mL of PBS (1×), making the stock solution. This solution was diluted in a cell culture medium (M199), in a 1:11 ratio. After exposing HUVECs to 50 μg/mL of extracellular histones for 4 h, and washing twice with PBS (1×) solution, the HUVECs were incubated for 3 h with the 1:11 MTT solution. After this time, 100 µL of DMSO was added to each well and incubated in the dark for 10 min with shaking. Finally, the absorbance of the plate was read at 590 nm, using the SpectraMax Plus 384 (Molecular Devices, San José, CA, USA).

The cell death determination was achieved by flow cytometry, using an Annexin V kit (Immunostep, Salamanca, Spain), following the manufacturer’s specifications. Briefly, after treating HUVECs for 4 h with 50 μg/mL of extracellular histones, cells were recovered together with the medium and resuspended in Annexin V buffer and stained with Annexin V/FITC and propidium iodide (PI). Cell viability, apoptosis, and necrosis were analyzed by flow cytometry in a FACS-Verse cytometer (Beckton Dickinson, San Jose, CA, USA) and determined by the Infinicyt software (Cytognos, Santa Marta de Tormes, Salamanca, Spain).

### 2.7. Western Blot

To obtain protein extracts, HUVECs were scraped with lysis buffer (20 mM Hepes, pH 7.4, 1% Triton X-100, 100 mM NaCl, 50 mM NaF, 10 mM β-glycerophosphate, 1 mM phenylmethylsulfonyl fluoride (PMSF), 1 mM sodium orthovanadate, and protease inhibitor cocktail (Roche Diagnostics, Mannheim, Germany)) and centrifuged for pellet (protein) recovery.

Western blotting (WB) was performed for the determination of protein relative levels: 10 μg of proteins were denatured using the sample buffer (Tris 40 mM, EDTA, bromophenol blue 0.01%, sucrose 40%, SDS 4%, and β-mercaptoethanol 10%) and heated to 95 ℃ for 5 min. Proteins were separated by sodium dodecyl sulfate/polyacrylamide gel electrophoresis (SDS-PAGE) and blotted onto nitrocellulose membranes (Whatman GmbH, Dassel, Germany). Proteins were incubated with specific primary antibodies, and detection was performed using their corresponding peroxidase-linked secondary antibodies (Supplementary Table S1). For loading control, β-actin was used. Finally, Luminol (ECL Western Blotting Detection Reagents, GE Healthcare, Hatfield, and Hertfordshire, UK) was added to the membrane for revealing proteins with an image reader LAS-4000 (GE Healthcare, Uppsala, Sweden). For signal density analysis, ImageJ software (NIH Image, National Institutes of Health, Bethesda, MD, USA) was used.

### 2.8. RNA Isolation and Purification and Quantitative Real-Time PCR Assay (RT-qPCR)

An miRNeasy Mini Kit (Qiagen, Hilden, Germany) was used for HUVEC RNA extraction and purification following the manufacturer’s instructions.

First, reverse transcription was performed using the High-Capacity cDNA Reverse Transcription Kit (Applied Biosystems, Foster City, CA, USA), with 200 ng of total RNA. The reaction was performed within a Mastercycler Eppendorf Thermocycler (Eppendorf, Hamburg, Germany).

For mRNA level determination, qPCR analysis was used. This assay was performed using the TaqMan Universal Mastermix (Thermo Fisher, Rockford, IL, USA) with the ABI Prism 7900 HT Fast Real-Time PCR System (Applied Biosystems). PCR conditions were 10 min at 95 °C for enzyme activation, followed by 40 two-step cycles (15 s at 95 °C; 1 min at 60 °C). The employed gene-specific primers and probes are described in Supplementary Table S2. The SDS software (Applied Biosystems. SDS, version 2.2.2; Waltham, Massachusetts, MA, USA) was employed for analyzing data according to the 2^−ΔΔCt^ method.

### 2.9. Statistical Analysis

Prism software v9.0.1 (GraphPad Software Inc. San Diego, CA, USA) was used for graphics and statistics, expressing the values as the mean ± SEM.

Before performing the different statistical analysis, an outlier test was performed, using the ROUT method and applying a 0.5% aggressiveness condition to discard outlier values. In the in vitro experiments, a nonparametric Mann-Whitney *t*-test was used. Significance was considered at * *p* ≤ 0.05, ** *p* ≤ 0.01, and *** *p* ≤ 0.001, as indicated in each case. For the studies performed in human blood samples, we used the Kolmogorov-Smirnov normality test to analyze the samples’ normality. To analyze the differences between two nonpaired groups, the nonparametric Mann-Whitney test was used, assuming a 0.05 level of significance. To analyze the statistically differences between the different groups of patients, the Kruskal-Wallis test was used, in addition to a post hoc test using Bonferroni correction for α (0.05/3). For correlation analysis between variables, Spearman’s rho test was used.

## 3. Results

### 3.1. Circulating and Citrullinated H3 Levels are Higher in Patients with Severe Phenotypes

**Figure 1:**
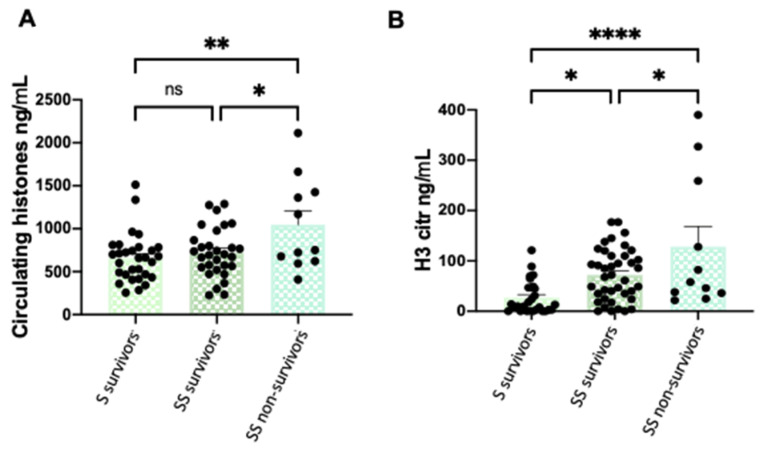
Circulating histone levels and H3cit levels differences among patient groups. (**A**) Variation in circulating histone levels among patient groups; (**B**) variation in H3cit levels among patient groups. These graphics show all sample times: first sample (during the first 24 h of patient admission in the ICU), second sample (after 3 days), third sample (after 5 days), and fourth sample (pre-discharge or before death). Data are expressed as the mean ± SEM; * *p* < 0.05, ** *p* < 0.01, **** *p* < 0.001, ns: not statistically significant. The lines at the top of the columns indicate differences between compared conditions. Abbreviations: ns, nonsignificant *p*-value; SS, septic shock; S: septic patients. Please note that H3citr levels were around 10 orders lower than the circulating histone concentration.

Circulating histones were measured by mass spectrometry in the 88 plasma samples obtained from patients with sepsis and SS with different clinical severity stages, showing the evolution of histones during their ICU stay (Figure 1A). The same 88 samples were used for measuring the circulating citrullinated (H3cit) levels along the ICU stay (Figure 1B). Our results indicate that higher circulating histone levels were found in those patients with worse clinical phenotypes (SS non-survivors) (Figure 1A). Similarly, higher levels of H3cit were found in patients with SS, with even higher levels in those who finally died.

**Figure 2:**
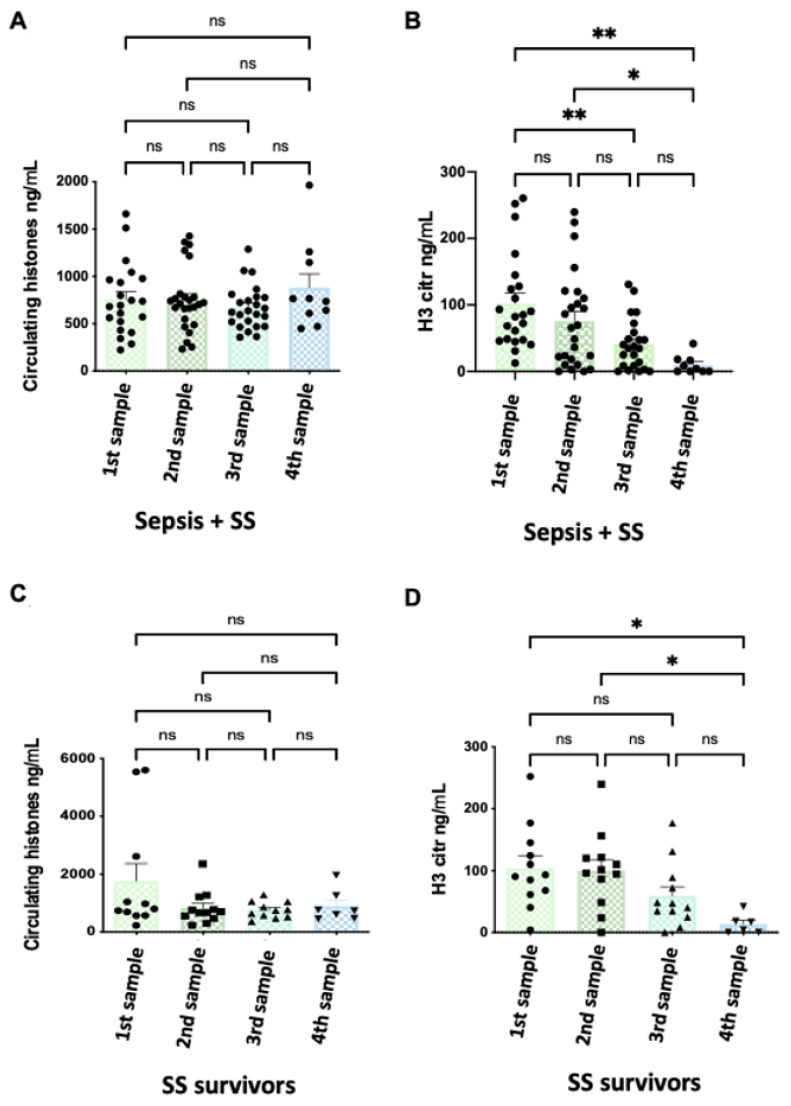
Circulating histone levels and H3cit variation along patients’ ICU stay in survivor patients. (**A**) Variation in circulating histone levels along the septic (sepsis and SS) patients’ ICU stay; (**B**) variation in H3cit levels along the septic (sepsis and septic shock) patients’ ICU stay. (**C**) Variation in circulating histone levels in SS survivor patients along ICU stay; (**D**) variation in H3cit levels in SS survivor patients along ICU stay. The X-axis shows the samples that were taken during the stay of patients in the ICU: first sample (during the first 24 h of patient admission in the ICU), second sample (after 3 days), third sample (after 5 days), and fourth sample (pre-discharge or before death). Data are expressed as the mean ± SEM; * *p* < 0.05, ** *p* < 0.01, ns: not statistically significant. The lines at the top of columns indicate differences between compared conditions. Abbreviations: ns, nonsignificant *p*-value.

Figure 2 shows the evolution of patients’ histone levels during their stay in the ICU. We did not find any statistical differences in circulating histones over time among patients when all septic patients (sepsis and SS) were analyzed together or individually (Supplementary Figure S2A,C). However, we found a significant reduction in H3cit along the ICU stay when analyzing sepsis and SS groups together, with much higher levels of H3citr at admission (first sample) than at the end of the ICU stay (fourth sample) (Figure 2B).

Then, we analyzed the H3cit histone levels along ICU stay in each separated group, and we observed significant reduction levels in the SS survivor group (Figure 2D), but not in the septic survivor or SS non-survivor groups (Supplementary Figure S2B,D).

### 3.2. Correlations between Clinical Features and Circulating and H3 Citrullinated Histones

**Figure 3:**
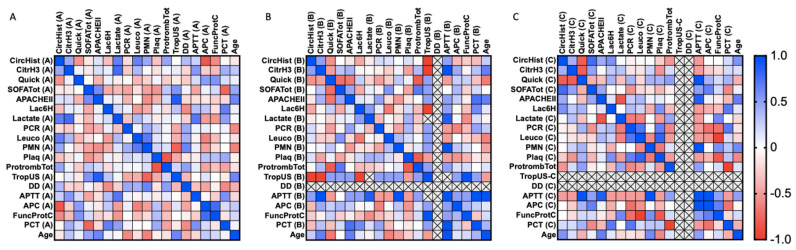
Heatmap representing sepsis survivor Spearman’s rank correlation coefficients (−1 to +1) among clinical variables analyzed. Red color indicates a negative correlation, and blue color indicates a positive correlation between compared parameters. (**A**) First sample obtained (during the first 24 h of patient admission in the ICU); (**B**) second sample obtained (after 3 days in the ICU); (**C**) third sample (after 5 days in the ICU). Abbreviations: CircHist: circulating histones; CitrH3: citrullinated H3; APC; activated protein C; SOFA: Sequential (sepsis-related) Organ Failure Assessment; APTT: activated partial thromboplastin time; PCR: reactive C protein; PCT: procalcitonin; PMN: polymorphonuclear cells; Plaq: platelets; Quick: quick SOFA; DD: D-dimer; TropUS: ultrasensitive troponin; FuncProtC: functional C protein; APACHE II: Acute Physiology and Chronic Health Disease Classification System II; Protromb Tot: total prothrombin; LAC6H: lactate after 6 h ICU entrance. The number of subjects analyzed was as follows: control (*n* = 10); intensive care unit (ICU) (*n* = 5); sepsis (*n* = 10); septic shock (SS) (*n* = 15).

**Figure 4:**
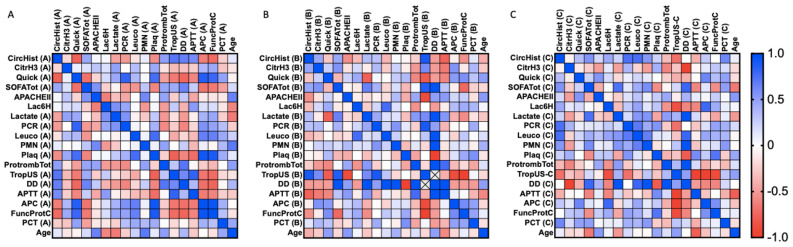
SS survivor heatmap representing Spearman’s rank correlation coefficients (−1 to +1) among clinical variables analyzed. Red color indicates a negative correlation, and blue color indicates a positive correlation between compared parameters. (**A**) First sample obtained (during the first 24 h of patient admission in the ICU); (**B**) second sample obtained (after 3 days in the ICU); (**C**) third sample (after 5 days in the ICU). Abbreviations: CircHist: circulating histones; CitrH3: citrullinated H3; APC; activated protein C; SOFA: Sequential (sepsis-related) Organ Failure Assessment; APTT: activated partial thromboplastin time; PCR: reactive C protein; PCT: procalcitonin; PMN: polymorphonuclear cells; Plaq: platelets; Quick: quick SOFA; DD: D-dimer; TropUS: ultrasensitive troponin; FuncProtC: functional C protein; APACHE II: Acute Physiology and Chronic Health Disease Classification System II; Protromb Tot: total prothrombin; LAC6H: lactate after 6 h ICU entrance. The number of subjects analyzed was as follows: control (*n* = 10); intensive care unit (ICU) (*n* = 5); sepsis (*n* = 10); septic shock (SS) (*n* = 15).

**Figure 5:**
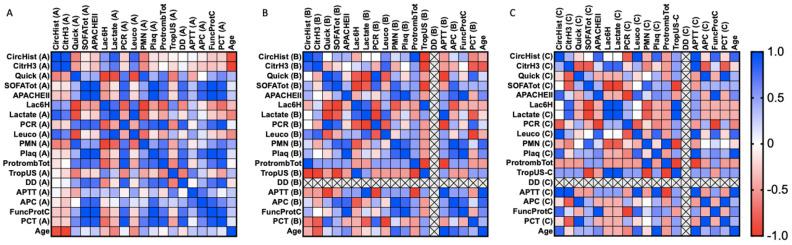
SS non-survivor heatmap representing Spearman’s rank correlation coefficients (−1 to +1) among clinical variables analyzed. Red color indicates a negative correlation, and blue color indicates a positive correlation between compared parameters. (**A**) First sample obtained (during the first 24 h of patient admission in the ICU); (**B**) second sample obtained (after 3 days in the ICU); (**C**) third sample (after 5 days in the ICU). Abbreviations: CircHist: circulating histones; CitrH3: citrullinated H3; APC; activated protein C; SOFA: Sequential (sepsis-related) Organ Failure Assessment; APTT: activated partial thromboplastin time; PCR: reactive C protein; PCT: procalcitonin; PMN: polymorphonuclear cells; Plaq: platelets; Quick: quick SOFA; DD: D-dimer; TropUS: ultrasensitive troponin; FuncProtC: functional C protein; APACHE II: Acute Physiology and Chronic Health Disease Classification System II; Protromb Tot: total prothrombin; LAC6H: lactate after 6 h ICU entrance. The number of subjects analyzed was as follows: control (*n* = 10); intensive care unit (ICU) (*n* = 5); sepsis (*n* = 10); septic shock (SS) (*n* = 15).

We provide a series of correlation analyses among clinical parameters and the circulating histone levels, as well as H3 citrullinated histones. Spearman’s rank correlation coefficients were calculated for clinical parameters in all cases studied (including sepsis and SS patients). The statistical values (correlation coefficient and *p*-value) are shown as a heatmap figure in Figure 3, Figure 4 and Figure 5, and the statistical values (*p*-value and correlation coefficient) are provided in Supplementary Tables S3–S11.

Focusing on the sepsis survivor group (Figure 3), several positive and negative correlations were found during the first 24 h after ICU admission (first sample) (Figure 3A) (Figure 3B,C), no correlations were found for circulating histones or H3cit.between circulating histones and circulating H3cit (Spearman *r* = 0.451, *p* = 0.05), as well as with quick SOFA (quick Sequential Organ Failure Assessment) (Spearman *r* = −0.525, *p* = 0.021), lactate (Spearman *r* = 0.668, *p* = 0.002), leukocytes (Spearman *r* = 0.658, *p* = 0.002), PMNs (polymorphonuclear cells) (Spearman r = 0.451, *p* = 0.05), total prothrombin (Spearman *r* = 0.47, *p* = 0.042), activated protein C (APC) (Spearman *r* = −0.83, *p* = 0.000), and ProtFuncC (functional protein C) (Spearman *r* = −0.574, *p* = 0.010). Similarly, some correlations were found between circulating H3cit and lactate (Spearman *r* = 0.772, *p* = 0.000), as well as APTT (active partial thromboplastin time) (Spearman *r* = 0.542, *p* = 0.016). Focusing on the second and third samples.

Regarding the SS survivor group (Figure 4), during the first 24 h after ICU admission (first sample) (Figure 4A), circulating H3cit correlated with lactate (Spearman *r* = 0.0486, p = 0.035), and circulating histones showed several positive and negative correlations among quick SOFA (Spearman *r* = −0.777, *p* = 0.000), SOFA Tot (Spearman *r* = 0.456, *p* = 0.050), lactate (Spearman *r* = −0.465, *p* = 0.045), PCR (reactive C protein) (Spearman *r* = −0.505, *p* = 0.027), platelets (Spearman *r* = −0.561, *p* = 0.012), total prothrombin (Spearman *r* = 0.498, p = 0.030), TropUS (ultrasensitive troponin) (Spearman *r* = 0.716, *p* = 0.001), DD (D-dimer) (Spearman *r* = 0.661, *p* = 0.002), APTT (Spearman *r* = 0.600, *p* = 0.007), APC (Spearman *r* = −0.632, *p* = 0.004), and ProtFuncC (Spearman *r* = −0.726, *p* = 0.000). In the second sample (Figure 4B), circulating histones correlated with APTT (Spearman *r* = −0.841, *p* = 0.036) and PCT (procalcitonin) (Spearman *r* = 0.714, *p* = 0.014), whereas circulating H3cit only correlated with total prothrombin (Spearman *r* = −0.663, *p* = 0.0226). In the third sample (Figure 4C), circulating histones correlated with PCR (Spearman *r* = 0.745, *p* = 0.017) and leukocytes (Spearman *r* = 0.758, *p* = 0.015), whereas circulating H3cit correlated with total prothrombin (Spearman *r* = −0.611, *p* = 0.049).

The most severe patient group, consisting of the SS non-survivor group (Figure 5), showed correlations at all timepoints studied. During the first 24 h after ICU admission (first sample) (Figure 5A), circulating histone levels correlated with circulating H3cit (Spearman *r* = 0.914, *p* = 0.000), quick SOFA (Spearman *r* = −0.614, *p* = 0.005), Lac6H (lactate at 6 h) (Spearman *r* = 0.542, *p* = 0.017), lactate (Spearman *r* = 0.851, *p* = 0.000), leukocytes (Spearman *r* = 0.803, *p* = 0.000), PMN (Spearman *r* = −0.619, *p* = 0.005), and age (Spearman *r* = −0.865, *p* = 0.000). At this time, circulating H3cit correlated with quick SOFA (Spearman *r* = −0.489, *p* = 0.034), Lac6H (Spearman *r* = 0.473, *p* = 0.041), lactate (Spearman *r* = 0.696, *p* = 0.001), leukocytes (Spearman *r* = 0.629, *p* = 0.004), PMN (Spearman *r* = −0.572, *p* = 0.011), and age (Spearman *r* = −0.954, *p* = 0.000). The second sample was taken 3 days after patient admission (Figure 5B). Circulating histones correlated with circulating H3cit (Spearman *r* = 0.8, *p* = 0.038), TropUS (Spearman *r* = 1, *p* = 0.022), and total prothrombin (Spearman *r* = 1, *p* = 0.022). Circulating citrullinated histones correlated with age (Spearman r = −0.8, *p* = 0.038), PCT (Spearman *r* = −0.8, *p* = 0.038), leukocytes (Spearman *r* = 0.8, *p =* 0.038), and TropUS (Spearman *r* = −1, *p* = 0.022). In the third sample (Figure 3C), which was taken on day 5 after admission, circulating histones correlated with age (Spearman *r* = 0.8, *p* = 0.038), APTT (Spearman *r* = 1, *p* = 0.022), PCR (Spearman *r* = –0.8, *p* = 0.038), leukocytes (Spearman *r* = 1, *p* = 0.001), platelets (Spearman *r* = 0.8, *p* = 0.0.038), ProtFuncC (Spearman *r* = 0.8, *p* = 0.038), and APACHE II (Spearman *r* = 0.8, *p* = 0.038), whereas the circulating H3cit correlated with APC (Spearman *r* = –0.8, *p* = 0.038), lactate (Spearman *r* = 0.8, *p* = 0.038), SOFA Tot (Spearman *r* = –0.8, *p* = 0.038), APTT (Spearman *r* = 1, *p* = 0.022), PCT (Spearman *r* = −0.8, *p* = 0.038), quick SOFA (Spearman *r* = −0.8, *p* = 0.038), and Lac6H (Spearman *r* = 0.8, *p* = 0.038).

### 3.3. Extracellular Citrullinated Histones Do Not Affect to Cellular Viability in HUVEC

Previous results have shown that H3cit is not able to cause cellular damage associated ETosis events [39]. In order to know the molecular mechanisms activated by citrullinated histones in those patients, an in vitro model of endothelial cells was used, since the endothelium is the first tissue in contact with histones when they are released into the bloodstream.

**Figure 6:**
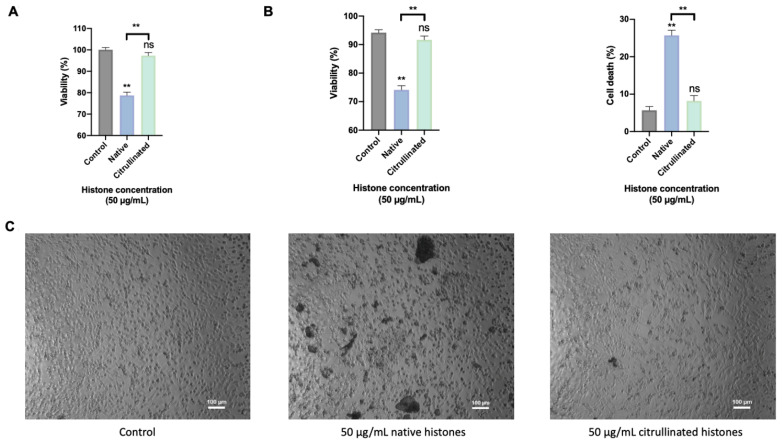
Cellular viability. (**A**) MTT method for cellular viability measurement. (**B**) Flow cytometry for viability/cell death measurement. (**C**) Optical microscopy for showing how HUVECs respond when exposed to extracellular histones. The scale of optical microscopy images is 100 μm. Data are expressed as the mean ± SEM from 3–5 independent experiments; ** *p* < 0.01, ns: not statistically significant, compared to control (histones 0μg/mL) unless the lines at the top of the columns indicate differences between compared conditions. Abbreviations: ns, nonsignificant *p*-value.

The cellular viability was analyzed using MTT experiments, flow cytometry, and optical microscopy in HUVEC exposed for 4 h to extracellular native and citrullinated histones. The MTT showed similar cell viability and cell death in control cells to HUVECs treated with citrullinated histones. However, cell death increased about threefold in HUVECs treated with native histones compared to cells treated with citrullinated histones (Figure 6A). These results were corroborated using flow cytometry by measuring the percentage of positive cells for propidium iodide and Annexin V (Figure 6B). Performing the analysis with optical microscopy, the results demonstrated that native histones induced an aggregation between histones and cells, which was not observed in cells exposed to extracellular citrullinated histones or in the control cells (Figure 6C).

### 3.4. Native Extracellular Histones Induce Oxidative Stress and Activate Antioxidant Response While Citrullinated Histones Do Not Alter the Oxidative Patterns

Previous studies have demonstrated that extracellular histones induce oxidative stress and the dysregulation of the antioxidant response, which was not able to counteract the ROS production induced by histones [40]. Because citrullinated histones were not able to induce cell death, we analyzed the antioxidant response to evaluate whether citrullinated histones were able to change the antioxidant response.

**Figure 7:**
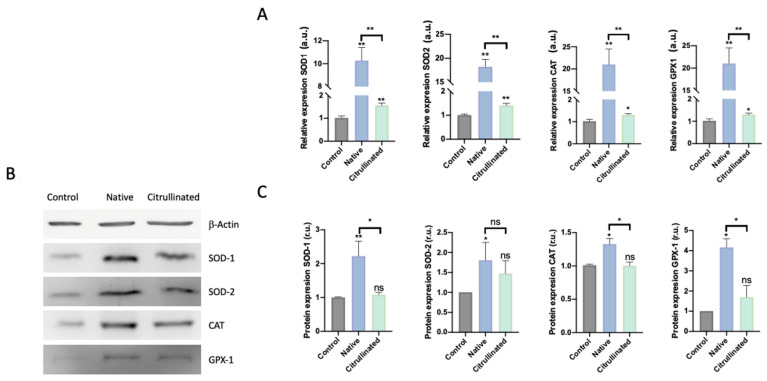
Oxidative stress and antioxidant response in HUVECs are challenged with 50μg/mL of extracellular histones (native and citrullinated). (**A**) Relative expression of antioxidant enzymes involved in the detoxification of superoxide (SOD1 and SOD2) and hydrogen peroxide (CAT and GPX1) evaluated by RT-qPCR in HUVECs treated with extracellular histones (native and citrullinated); (**B**) representative Western Blot showing the protein levels of the antioxidant enzymes CuZnSOD (SOD-1), MnSOD (SOD-2), catalase (CAT), and glutathione peroxidase 1 (Gpx-1); (**C**) densitometry of 3–5 independent Western blot experiments showing the relative amount of the antioxidant enzymes in each of the analyzed conditions. Data are expressed as the mean ± SEM from 3–5 independent experiments; * *p* < 0.05, ** *p* < 0.01, compared to control (histones 0 μg/mL) unless the lines at the top of the columns indicate differences between compared conditions. Abbreviations: ns, nonsignificant *p*-value; a.u., arbitrary units; r.u., relative units. The lines at the top of the columns indicate differences between compared conditions.

HUVECs exposed to extracellular native histones showed a statistically significant overexpression, in both mRNA and protein levels, of antioxidant enzymes. However, those cells exposed to citrullinated histones only showed the overexpression of the mRNA (Figure 7A) but not in the protein levels of the antioxidant enzymes (Figure 7B,C).

### 3.5. Prostanoid Biosynthesis is Altered by Extracellular Histones in HUVECs

Prostanoids are molecules produced and released by HUVECs, implicated in the contraction and relaxation of endothelium by acting at the smooth muscle level [41]. In this regard, we analyze whether extracellular histones and citrullinated ones can modify the normal function of the endothelium.

**Figure 8:**
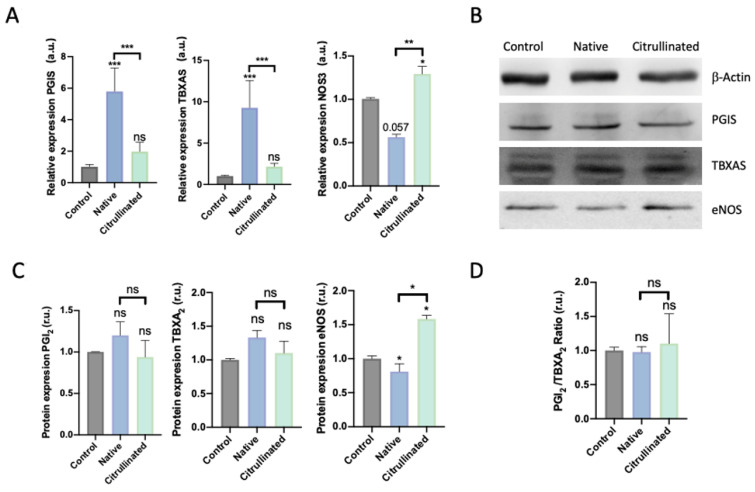
Prostanoids and endothelial response in HUVECs challenged with 50 μg/mL of extracellular histones (native and citrullinated). (**A**) Relative expression of the enzymes involved in the synthesis of prostanoids and eNOS determined by RT-qPCR; (**B**) a representative Western blot showing the protein levels of PGIS, TBXAS, and oxide nitrate synthase; (**C**) densitometry of 3–5 independent Western blot experiments showing the relative amount of proteins (PGI2, TBXA2, and the oxide nitrate synthase) and (**D**) PGI2/TBXA2 ratio. Data are expressed as the mean ± SEM from 3–5 independent experiments; * *p* < 0.05, ** *p* < 0.01, *** *p* < 0.001, compared to control (histones 0 μg/mL) unless the lines at the top of the columns indicate differences between compared conditions. Abbreviations: ns, nonsignificant *p*-value; a.u., arbitrary units; r.u., relative units.

When HUVECs were exposed to extracellular histones, both prostacyclin synthase (PGIS) and thromboxane synthase (TBXAS) dramatically increased their expression (Figure 8A). This overexpression was not observed when HUVECs were exposed to citrullinated histones. Nevertheless, the PGI_2_/TBXA_2_ ratio was not affected when cells were exposed to histones, whether native or citrullinated (Figure 8D). Surprisingly, the endothelial nitric oxide synthase (eNOS) expression levels decreased after HUVECs were challenged with extracellular native histones but increased in HUVECs challenged with citrullinated histones (Figure 8B). eNOS protein levels were decreased in HUVECs exposed to native histones (Figure 8B,C); conversely, the levels of eNOS were increased in those exposed to citrullinated histones (Figure 8C).

### 3.6. Extracellular Histones Induce Molecular Pathways of Inflammation and Modulate Pathways of Prostanoidbiosynthesis

Cyclooxygenases (COX) are master regulators of the prostanoid pathways [42]. COX isoforms have been postulated as important mediators of different inflammatory proteins such as IL-6 [43]. Specifically, COX-1 modulates thromboxane (TBXA_2_) synthesis, while COX-2 is responsible for prostacyclin A_2_ (PGI_2_) synthesis, and this isoform is related to inflammatory phenotypes [43]. In this regard, IL-6 is one of the first cytokines released during inflammation, including the sepsis hyperinflammatory cascade [44]. Inflammation in the endothelium has been related to the immune response, where ICAM-1, VCAM-1, and e-SEL play a central role, mediating the leucocyte rolling (e-SEL) and the transendothelial migration (I-CAM1 and V-CAM1) [45].

**Figure 9:**
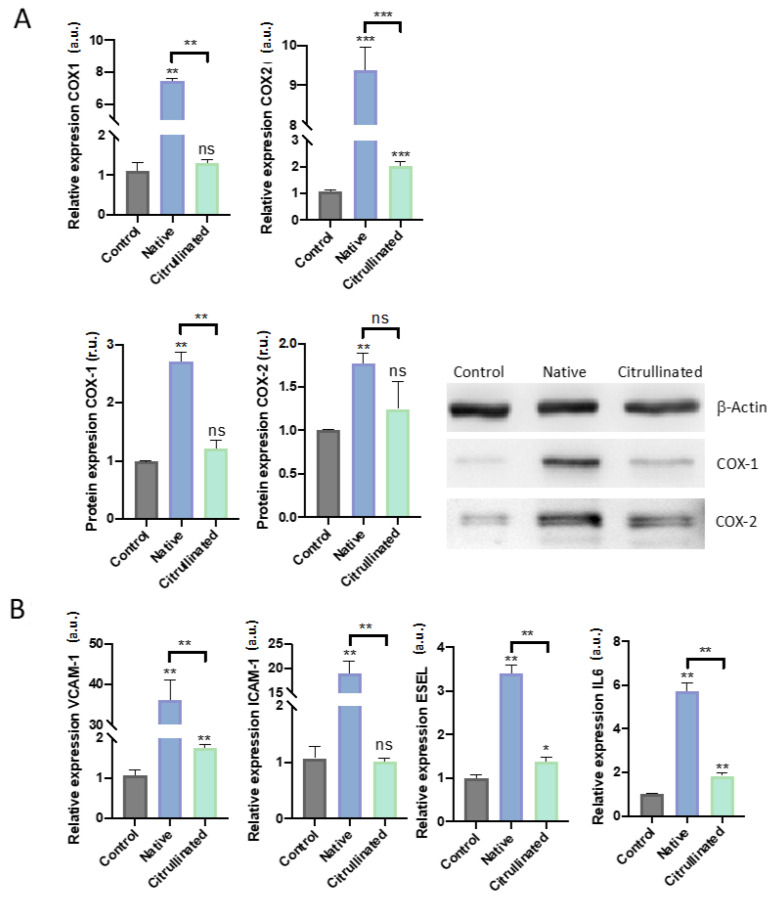
Immune response in HUVECs challenged with 50 μg/mL of extracellular histones (native and citrullinated). Data are expressed as the mean ± SEM from 3–5 independent experiments; * *p* < 0.05, ** *p* < 0.01, *** *p* < 0.001. (**A**) Relative expression evaluated by RT-qPCR and WB for regulators of the prostanoid pathway, COX-1, and COX-2; (**B**) Relative expression evaluated by RT-qPCR of the mediators of endothelial adhesion (VCAM-1, ICAM-1, and ESEL) and the proinflammatory IL-6. Statistics compared to control (histones 0 μg/mL) unless the lines at the top of the columns indicate differences between compared conditions. Abbreviations: ns, nonsignificant *p*-value; a.u., arbitrary units; r.u., relative units.

Our results demonstrated that HUVECs exposed to extracellular histones in native conditions induce COX-1 expression (Figure 9A), which correlated with prostanoid levels. Interestingly, the citrullinated histones did not alter the expression of COX-1 (Figure 9A). Conversely to the results obtained for COX-1, we found an increment in COX-2 expression when cells were exposed to both native and citrullinated histones (Figure 9A).

Regarding IL-6 expression, HUVECs exposed to extracellular native histones expressed high levels of this cytokine, as did HUVECs exposed to citrullinated histones but to a lesser extent (Figure 9B). Similarly, HUVECs exposed to extracellular histones increased the expressions levels of ICAM-1, VCAM-1, and E-SEL (Figure 9B), while HUVECs exposed to extracellular citrullinated histones increased the levels of VCAM-1 and ESEL to a lesser extent and did not alter the expression levels of ICAM-1 (Figure 9B).

## 4. Discussion

During sepsis, the invading pathogens act as PAMPs and stimulate the release of DAMPs, which promote and exacerbate the immune system response, causing a cytokine storm that damages body organs [46]. The most described DAMPs during sepsis are the nuclear content, including DNA and histones [47]. They are released into the bloodstream mainly through cell death mechanisms, such as apoptosis or necrosis, and through innate immune processes such as ETosis [48], a process which requires the nuclear citrullination of H3 [49,50]. ETosis modulates the overactivation of the immune system during sepsis; hence, it would be expected to find a high amount of citrullinated histones (especially H3cit) into the bloodstream in the first stages of sepsis, just when the innate immunity is occurring [51,52]. However, we found that, despite levels of circulating histones remaining elevated during the stay of patients in the ICU, the levels of circulating H3cit decreased along the septic process. This means that ETosis decreases over time, either because its activation occurs only at the beginning of sepsis, when the body needs protection against pathogens, or because of the immune paralysis that can occur during sepsis [53,54,55]. Another likely explanation could be immunosuppression, which also takes place during sepsis, as other authors have postulated [53,56]. In this regard, some reports have described two different immune phases during sepsis, starting with a hyper-inflammatory phase (also called cytokine storm phase), and continuing with a hypo-inflammatory or immune paralysis phase, leading to immunosuppression in those patients who survive sepsis [53,54,55] (as reviewed by Boomer et al., 2014 [53] and Beltrán-Garcia et al., 2020 [56]).

We propose that the main source of circulating histones during the early sepsis stage is not due to the ETosis process, because the higher levels of histones found were not citrullinated. In fact, we quantified almost tenfold higher levels of circulating histones than H3cit. In addition, we showed that the patient group with higher histone levels (both circulating histones and H3cit) was the SS non-survivor group. Nevertheless, the number of samples analyzed for the SS non-survivor group was low; thus, it is possible that the statistical analysis was not robust enough, which is a limitation of our study. In SS, we found that high levels of circulating histones correlated with worst clinical characteristics and poor prognosis [57]. Likewise, H3cit levels are higher in this group because they may counteract the infection more efficiently, activating those extreme mechanisms of innate immune defense that are mediated by ETosis [58].

Interestingly, citrullinated histones showed a clear tendency to decrease throughout the patients’ ICU stay, suggesting that there exist strong differences in the immune response between septic and SS patients. In this regard, Gogos et al., recently found differences between the early status of the innate and adaptive immune system between sepsis and SS patients, and those differences were related to the infection source [59]. Regarding innate and adaptive immune activation, our group also showed that during the first stages of sepsis, adaptive immunity is not completely active [60]. In line with these observations, we propose that, during the first stages of sepsis, innate immunity is overexpressed in comparison with the later stages of sepsis, especially in the SS patient group. In this regard, analyzing the correlations between circulating histones, circulating H3cit, and clinical phenotypes, we found that SS non-survivors showed a strong positive correlation between circulating histones and H3cit. Moreover, this correlation disappeared during the ICU stay, probably due to the decrease in H3cit levels.

Many authors have related the elevated plasma histone levels to the severity of sepsis, due to their ability to cause cellular damage and inflammation [47,61]. In this regard, we found that circulating histones positively correlated with lactate in the first samples, indicating that circulating histones correlate with patient severity, as described previously by other authors [47,61,62].

Histones have also been related to coagulopathies [63], specifically histone H3 [64]. In our results, we observed some correlations indicating this clinical feature in the analyzed patients. Specifically, in second sample, we found a clear positive correlation between circulating histones and total prothrombin. In addition, circulating H3 has been related to the prolonged prothrombin time in ICU septic patients where coagulation is activated and has an increased risk of death [63]. These results are in agreement with the correlation that we found in the SS non-survivor group with APTT in third sample, which was the patient group with the worst outcome. Furthermore, we found a correlation in the third sample between H3cit and platelets which could involve the endothelium. We propose that platelets are unable to adhere to the endothelium, thus requiring more time to develop a clot. This matches with the positive correlation between H3cit and functional protein C, which is also related to APTT.

As deduced from these results, circulating histones are closely related to coagulation in which endothelium dysfunction takes an important role. Our group recently explained how endothelial dysfunction is triggered by histones through endothelial pyroptosis, thus contributing to inflammatory phenotypes and endothelial cell death [40,65]. Many authors have related ETosis of neutrophils (NETosis) to thrombus [66], but we did not find any correlation between H3cit and coagulopathies in septic patients. However, H3cit negatively correlated with Trop US, in SS non-survivor patients, and with APC. These results suggest that NETosis may induce coagulopathies in septic patients, and this process may be a consequence of the release of other proteins during the NETosis process, such as PAD proteins [67,68,69,70,71], not only because of the release of H3cit. In fact, we found using in vitro experiments that H3cit has a protective role, especially in comparison with circulating histones.

It is noteworthy, survivors of sepsis are prone to suffer from long-term comorbidities, and they have an increased risk of dying within the next 2 years after surviving a sepsis episode [72,73]. This is important because many correlations related to coagulopathies have been observed in the SS survivor group, especially during the first 24 h after ICU admission (first sample). We observed that circulating histones correlated negatively with platelets and with anticoagulant characteristics (APC and functional C protein) and coupled positively with procoagulant factors (total prothrombin, ultrasensitive prothrombin, DD, and APTT). Exceptionally, APTT correlated negatively in second sample, contributing to the onset of disseminated intravascular coagulopathies (DICs). Some authors have described DIC associated with sepsis, and this may be the reason for those correlations that we observed since it is related to low platelet levels, high thrombin levels, and a hypercoagulation status [74,75].

We also observed relevant correlations between histones and leukocytes, but only the SS non-survivor group showed correlations with PMN. Specifically, negative correlations were shown between circulating histones and H3cit with PMN, which can be explained by the high amount of circulating H3cit in this patient group. Circulating histone levels correlated positively with leukocytes for the first and last time in this group, and, in the first sample and second samples, leukocytes correlated with H3cit, indicating that the immune system is active and trying to counteract the infection.

The sepsis survivor group showed different correlations during the first 24 h after ICU admission (first sample). Circulating histones correlated with lactate, indicating that the damage affecting the organs of those patients correlates with leukocyte number and PMN, indicating that the immune system is being activated. A weak correlation was found between circulating histones and total prothrombin, but strong negative correlations were found with anticoagulant features, such as APC and functional protein C, indicating that the anticoagulant response is counteracting the procoagulant response, which is reinforced by the fact that no correlations were observed in the second or third samples, probably because septic patients are in a better clinical stage than those in the SS group.

NETosis damage the patients’ body [76], and circulating histones contribute to organ failure [61,77] and endothelial cell damage [65]; therefore, after analyzing the correlations between H3cit and clinical features, we focused on analyzing the molecular mechanisms activated by H3cit in endothelial cells. The endothelium is one of the first affected tissues during sepsis [22,78], as it is involved in the recovery of the blood vessels in the places where DAMPs and PAMPs are released. In this regard, we observed that native but not citrullinated histones induced cell death and oxidative stress in HUVECs. Moreover, eNOS expression was downregulated in HUVECs treated with native histones and was overexpressed after citrullinated histone treatment. This differential modulation of eNOS and endothelial viability by native and citrullinated histones reinforce the idea that, even though NETosis has been related to thrombotic events, the citrullination of histones may not contribute to thrombosis. In a similar way, native histones also affect the prostanoid pathway increasing the expression of cyclooxygenases, prostacyclin, and thromboxane synthase which, together with NO, affect vascular homeostasis. Subsequently, while native histones deregulate the endothelial cells, as demonstrated previously by our group [33,34,65], citrullinated histones do not disturb the endothelial function, which could explain why the clinical correlations we found were worse in circulating histones than in H3cit.

Other authors have associated the release of histones to the bloodstream with inflammation [79]. We observed that both native and citrullinated histones induced the activation of IL-6, although native histones produced a major increase of IL-6 than the H3cit. Moreover, VCAM-1, ICAM-1, and E-SEL, which are involved in the immune response mediated by endothelium, are overexpressed by both native and citrullinated histones. Thus, we propose that citrullinated histones have the ability to induce an immune response as they are released mainly during ETosis, but may not cause host damage, confirming the hypothesis that citrullinated histones are less cytotoxic than native histones.

In summary, we can conclude that citrullinated histones are released mainly by the innate immune system and reach a maximum concentration at the initial stage of sepsis and decrease during the time of ICU stay. In addition, despite histones and NETosis events being related to coagulopathies, H3cit may not cause tissue damage. Lastly, we can conclude that high levels of circulating histones are associated with poor prognosis, regardless of H3cit levels.

## 5. Patents

J.L.G.-G., N.C., and F.V.P. are inventors of patents related to the detection of circulating histones (EP3535587B1) and activated protein C (EP21382173.9) by mass spectrometry.

## Figures and Tables

**Table 1:** Peptides selected for MRM-MS quantification.

Protein	Peptide
Histone H2B	LLLPGELAK
Histone H3	STELLIR
Histone H4	DNIQGITKPAIR and VFLENVIR

## Data Availability

Not applicable.

## Supplementary Materials

The following supporting information can be downloaded at https://www.mdpi.com/article/10.3390/cells11244070/s1: Figure S1. In vitro citrullination; Table S1. Primary and secondary antibodies employed in WB; Table S2. Probes employed in RT1-PCR analysis; Figure S2. Circulating and citrullinated levels obtained in septic survivor patients and SS non-survivors; Table S3. Spearman’s correlations in sepsis survivor group, first sample collected; Table S4. Spearman’s correlations in sepsis survivor group, second sample collected; Table S5. Spearman’s correlations in sepsis survivor group, third sample collected; Table S6. Spearman’s correlations in SS survivor group, first sample collected; Table S7. Spearman’s correlations in SS survivor group, second sample collected; Table S8. Spearman’s correlations in SS survivor group, third sample collected; Table S9. Spearman’s correlations in SS non-survivor group, first sample collected; Table S10: Spearman’s correlations in SS non-survivor group, second sample collected; Table S11. Spearman’s correlations in SS non-survivor group, third sample collected.
