# Supplementary material for: Histone Citrullination Mediates a Protective Role in Endothelium and Modulates Inflammation
Source: Cells. 2022 Dec 15;11(24):4070. doi: 10.3390/cells11244070 (PMC9777278; doi:10.3390/cells11244070)
Supplement: Supplementary file 1 [file cells-11-04070-s001.zip › cells-2004413-supplementary.pdf]

Supplementary Material and Results

Histone citrullination mediates a protective role in endothelium and modulates inflammation

Rebeca Osca-Verdegal,[1,2] Jesús Beltrán-García,[3] Ana B. Paes,[2,4] Elena Nacher-Sendra,[2,4] Susana Novella,[2,4] Carlos Hermenegildo,[2,4] Nieves Carbonell,[4,5] José Luis García-Giménez[1,2,4,6*], Federico V. Pallardó,[1,2,4]

[1] Centro de Investigación Biomédica en Red de Enfermedades Raras (CIBERER). Instituto de Salud Carlos III. Madrid, Spain, 28029

[2] Departamento de Fisiología. Facultad de Medicina y Odontología. Universitat de València. València, Spain, 46010

[3] Department of Medicine, Division of Regenerative Medicine, University of California, San Diego, La Jolla, CA, USA

[4] Instituto de Investigación Sanitaria INCLIVA. Valencia, Spain, 46010

[5] Hospital Clínic Universitari de València. Valencia, Spain, 46010

[6] EpiDisease S.L. (Spin-Off CIBER-ISCIII), Parc Científic de la Universitat de València, Paterna, Valencia, Spain

Corresponding autor:

José Luis García-Giménez

j.luis.garcia@uv.es

Supplementary Materials

2.5. Histone extraction, purification and in vitro citrullination

Histone citrullination has induced using the PAD Cocktail and different extracellular histone concentrations. The citrullination levels were analyzed by WB and demonstrated that the best condition for citrullination induction was the ratio 1 to 1, same quantity of histones than PAD cocktail (Figure 1A and 1B). Because it is known that almost all histone isoforms can be citrullinated, a WB was performed to confirm which histones are being citrullinated. The figure 1C and 1D shows that histone H4, H2A and H2B can be citrullinated. Figure 1E confirms that the most citrullinated histone was histone H3 as expected.

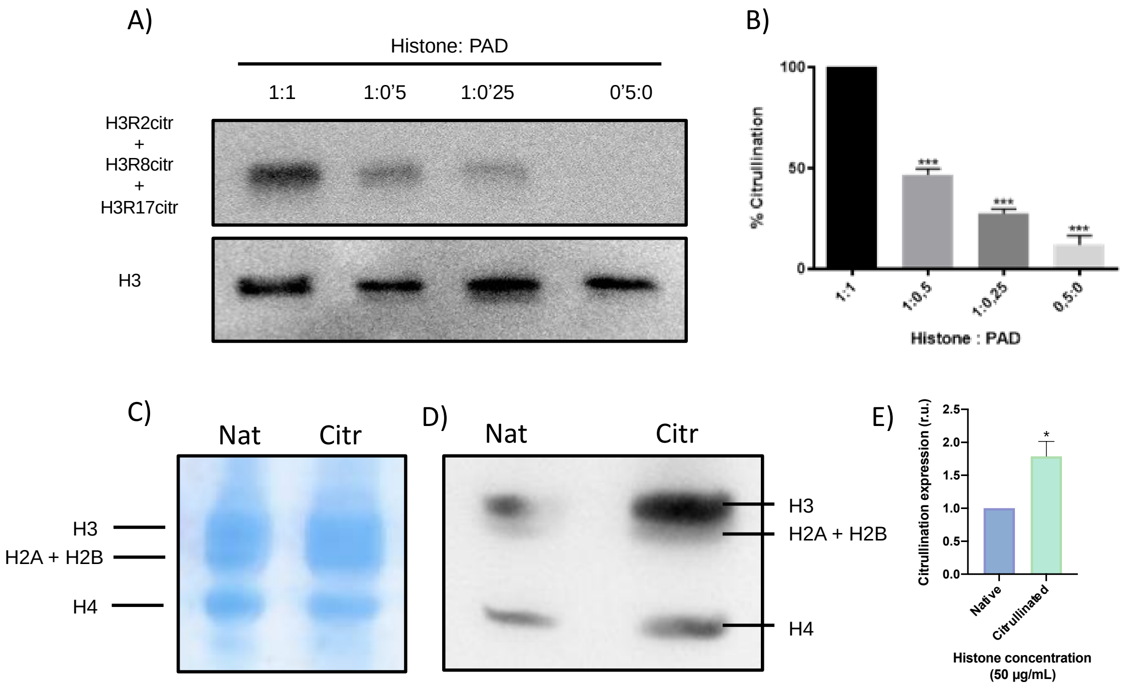

Supplementary Figure S1. *In vitro* citrullination. A) WB showing the citrullination obtained using different ratios of Histone:PAD. B) Quantification of citrullination obtained in A. C) Load control of histone citrullination. D) WB obtained for histone citrullination measurements. E) Histone citrullination quantification. Abbreviatures: PAD: Peptidylarginine Deiminases; Nat: Native histones.

2.7. Western Blot

Supplementary Table S1. Primary and secondary antibodies employed in WB.

	Antibodies	Reference
Primary antibodies	GPX1	Abcam, ab22604
	Catalase	Sigma-Aldrich, c0979
	MnSOD	Abcam, ab13533
	Cu/ZnSOD	Abcam, ab13498
	PGIS	Santa Cruz Biotechnology, sc-20933
	TXAS	Santa Cruz Biotechnology, sc-79181
	eNOS	Santa Cruz Biotechnology, sc-653
	COX-1	Santa Cruz Biotechnology, sc-19998
	COX-2	Santa Cruz Biotechnology, sc-19999
	β-actin	Sigma-Aldrich, a1978
Secondary antibodies	anti-mouse	Sigma-Aldrich, NA931V
	anti-goat	Santa Cruz Biotechnology, sc2020
	anti-rabbit	Sigma-Aldrich, NA934V

2.8. RNA isolation and purification and quantitative real-time PCR assay (qRT-PCR)

Supplementary Table S2. Probes employed in RTq-PCR analysis.

Probes	Reference
PGIS-2	Hs00919949_m1, Life Technologies
TBXAS	Hs01022706_m1, Life Technologies
NOS-3	Hs01574665_m1, Life Technologies
V-CAM-1	Hs01003372_m1, Life Technologies
I-CAM-1	Hs00164932_m1, Life Technologies
SEL-E	Hs00174057_m1, Life Technologies
COX-1	Hs00377726_m1, Life Technologies
COX-2	Hs00153133_m1, Life Technologies
IL-1α	Hs00174092_m1, Life Technologies
IL-1β	Hs01555410_m1, Life Technologies
IL-6	Hs00174131_m1, Life Technologies
IL-18	Hs01038788_m1, Life Technologies
GAPDH	Hs02758991_g1, Life Technologies

Supplementary Results

3.1. Circulating and citrullinated H3 levels are higher in patients with severe phenotypes

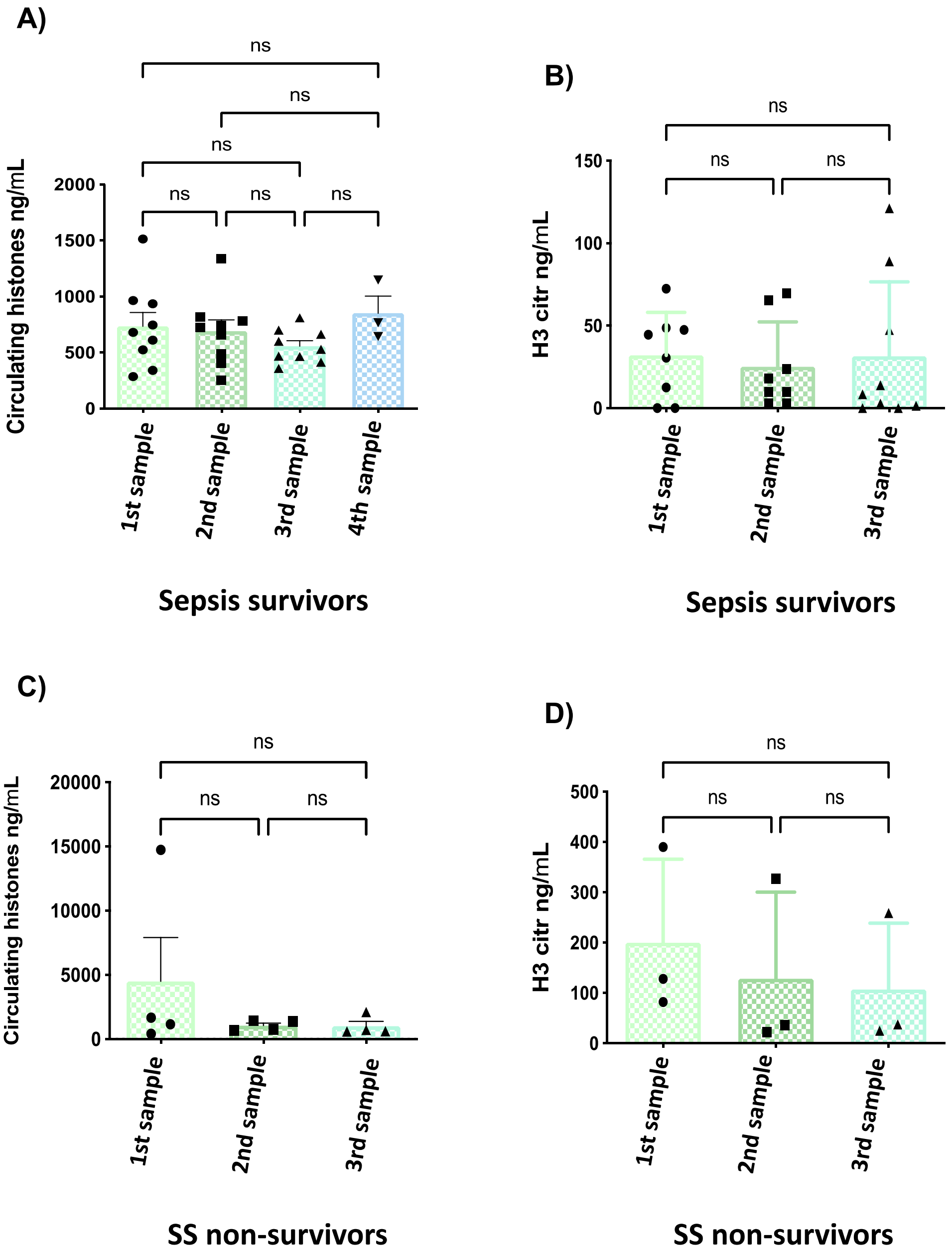

Supplementary Figure S2. Circulating and citrullinated levels obtained in septic survivors' patients and SS non-survivors. A and B) Circulating histones evolution along the time in the different patients' groups. H3 citrullinated evolution (C-D) along the time in the different patients' groups. The X-axis shows the samples that were taken during the stay of patients in the ICU: 1st sample (during the first 24-hours of patient admission in the ICU), 2nd sample (after 3 days); 3rd sample (after 5 days), 4th sample (pre-discharge or before death). Data are expressed as mean±SEM. ns: not statistically significant. The lines at the top of columns indicate differences between compared conditions. Abbreviation: ns, non-significant p-value.

3.2. Correlations between clinical features and circulating and H3 citrullinated histones

Supplementary Table S3. Spearman's correlations in sepsis survivor's group, 1st sample collected.

		Age	CircHist-A	CitrH3-A	PCA-A	Lactate-A	SOFATot-A	APTT-A	PCR-A	PCT-A	LEUCO-A	PMN-A	Plaq-A	Quick-A	DD-A	TropUS-A	FuncProtC	APACHEII	PROTOTAL	LAC6H
**Age**	**correlation coefficient**	1,000	-0,119	0,309	0,282	0,032	0,470	-0,205	-0,589	0,068	-0,168	-0,616	-0,088	0,086	-0,404	0,635	0,205	0,632	-0,158	0,088
	**significance**		0,627	0,198	0,241	0,898	0,042	0,399	0,008	0,781	0,491	0,005	0,721	0,726	0,087	0,003	0,399	0,004	0,519	0,721
**CircHist-A**	**correlation coefficient**	-0,119	1,000	0,451	-0,830	0,668	-0,125	0,244	-0,186	-0,056	0,658	0,451	-0,305	-0,525	0,349	-0,035	-0,574	0,156	0,470	-0,325
	**significance**	0,627		0,053	0,000	0,002	0,611	0,314	0,446	0,819	0,002	0,053	0,204	0,021	0,143	0,887	0,010	0,523	0,042	0,175
**CitrH3-A**	**correlation coefficient**	0,309	0,451	1,000	-0,251	0,772	-0,125	0,542	-0,279	0,421	0,372	0,035	-0,154	0,074	-0,214	0,105	-0,346	0,175	0,063	0,100
	**significance**	0,198	0,053		0,300	0,000	0,611	0,016	0,247	0,073	0,117	0,887	0,528	0,764	0,379	0,668	0,147	0,473	0,797	0,684
**PCA-A**	**correlation coefficient**	0,282	-0,830	-0,251	1,000	-0,500	0,075	-0,053	0,091	0,189	-0,618	-0,481	0,323	0,514	-0,456	0,153	0,775	-0,125	-0,453	0,295
	**significance**	0,241	0,000	0,300		0,029	0,759	0,831	0,710	0,437	0,005	0,037	0,178	0,024	0,050	0,533	0,000	0,611	0,052	0,221
**Lactate-A**	**correlation coefficient**	0,032	0,668	0,772	-0,500	1,000	-0,360	0,716	-0,312	0,504	0,356	0,147	-0,098	-0,098	-0,242	0,061	-0,528	0,089	0,079	0,191
	**significance**	0,898	0,002	0,000	0,029		0,130	0,001	0,193	0,028	0,135	0,547	0,689	0,689	0,318	0,803	0,020	0,716	0,748	0,433
**SOFATot-A**	**correlation coefficient**	0,470	-0,125	-0,125	0,075	-0,360	1,000	-0,453	-0,465	-0,756	-0,170	-0,472	-0,526	-0,002	0,068	0,554	0,198	0,596	0,295	-0,425
	**significance**	0,042	0,611	0,611	0,759	0,130		0,052	0,045	0,000	0,486	0,041	0,021	0,994	0,781	0,014	0,416	0,007	0,221	0,070
**APTT-A**	**correlation coefficient**	-0,205	0,244	0,542	-0,053	0,716	-0,453	1,000	0,074	0,575	-0,065	0,091	-0,102	0,189	-0,321	-0,025	-0,247	-0,168	0,114	0,337
	**significance**	0,399	0,314	0,016	0,831	0,001	0,052		0,764	0,010	0,792	0,710	0,679	0,437	0,180	0,920	0,307	0,491	0,642	0,158
**PCR-A**	**correlation coefficient**	-0,589	-0,186	-0,279	0,091	-0,312	-0,465	0,074	1,000	0,074	0,056	0,435	0,233	-0,044	0,454	-0,432	0,012	-0,386	0,058	0,096
	**significance**	0,008	0,446	0,247	0,710	0,193	0,045	0,764		0,764	0,819	0,063	0,336	0,858	0,051	0,065	0,960	0,103	0,814	0,694
**PCT-A**	**correlation coefficient**	0,068	-0,056	0,421	0,189	0,504	-0,756	0,575	0,074	1,000	-0,002	0,005	0,440	0,181	-0,574	-0,112	-0,130	-0,196	-0,463	0,675
	**significance**	0,781	0,819	0,073	0,437	0,028	0,000	0,010	0,764		0,994	0,983	0,059	0,459	0,010	0,647	0,596	0,420	0,046	0,002
**LEUCO-A**	**correlation coefficient**	-0,168	0,658	0,372	-0,618	0,356	-0,170	-0,065	0,056	-0,002	1,000	0,695	0,144	-0,360	0,409	-0,398	-0,335	-0,030	0,042	-0,502
	**significance**	0,491	0,002	0,117	0,005	0,135	0,486	0,792	0,819	0,994		0,001	0,557	0,130	0,082	0,091	0,161	0,904	0,864	0,029
**PMN-A**	**correlation coefficient**	-0,616	0,451	0,035	-0,481	0,147	-0,472	0,091	0,435	0,005	0,695	1,000	0,228	-0,233	0,637	-0,768	-0,191	-0,561	0,163	-0,421
	**significance**	0,005	0,053	0,887	0,037	0,547	0,041	0,710	0,063	0,983	0,001		0,348	0,336	0,003	0,000	0,433	0,012	0,505	0,073
**Plaq-A**	**correlation coefficient**	-0,088	-0,305	-0,154	0,323	-0,098	-0,526	-0,102	0,233	0,440	0,144	0,228	1,000	0,058	-0,146	-0,435	0,340	-0,393	-0,835	0,081
	**significance**	0,721	0,204	0,528	0,178	0,689	0,021	0,679	0,336	0,059	0,557	0,348		0,814	0,552	0,063	0,154	0,096	0,000	0,743
**QuickA**	**correlation coefficient**	0,086	-0,525	0,074	0,514	-0,098	-0,002	0,189	-0,044	0,181	-0,360	-0,233	0,058	1,000	-0,265	-0,112	0,561	-0,377	-0,260	0,084
	**significance**	0,726	0,021	0,764	0,024	0,689	0,994	0,437	0,858	0,459	0,130	0,336	0,814		0,273	0,647	0,012	0,111	0,283	0,732
**DD-A**	**correlation coefficient**	-0,404	0,349	-0,214	-0,456	-0,242	0,068	-0,321	0,454	-0,574	0,409	0,637	-0,146	-0,265	1,000	-0,365	-0,089	-0,195	0,504	-0,579
	**significance**	0,087	0,143	0,379	0,050	0,318	0,781	0,180	0,051	0,010	0,082	0,003	0,552	0,273		0,124	0,716	0,424	0,028	0,009
**TropUS-A**	**correlation coefficient**	0,635	-0,035	0,105	0,153	0,061	0,554	-0,025	-0,432	-0,112	-0,398	-0,768	-0,435	-0,112	-0,365	1,000	-0,009	0,874	0,223	0,312
	**significance**	0,003	0,887	0,668	0,533	0,803	0,014	0,920	0,065	0,647	0,091	0,000	0,063	0,647	0,124		0,972	0,000	0,359	0,193
**FuncProtC**	**correlation coefficient**	0,205	-0,574	-0,346	0,775	-0,528	0,198	-0,247	0,012	-0,130	-0,335	-0,191	0,340	0,561	-0,089	-0,009	1,000	-0,242	-0,316	-0,170
	**significance**	0,399	0,010	0,147	0,000	0,020	0,416	0,307	0,960	0,596	0,161	0,433	0,154	0,012	0,716	0,972		0,318	0,188	0,486
**APACHEII**	**correlation coefficient**	0,632	0,156	0,175	-0,125	0,089	0,596	-0,168	-0,386	-0,196	-0,030	-0,561	-0,393	-0,377	-0,195	0,874	-0,242	1,000	0,193	0,081
	**significance**	0,004	0,523	0,473	0,611	0,716	0,007	0,491	0,103	0,420	0,904	0,012	0,096	0,111	0,424	0,000	0,318		0,429	0,743
**PROTOTAL**	**correlation coefficient**	-0,158	0,470	0,063	-0,453	0,079	0,295	0,114	0,058	-0,463	0,042	0,163	-0,835	-0,260	0,504	0,223	-0,316	0,193	1,000	-0,182
	**significance**	0,519	0,042	0,797	0,052	0,748	0,221	0,642	0,814	0,046	0,864	0,505	0,000	0,283	0,028	0,359	0,188	0,429		0,455
**LAC6H**	**correlation coefficient**	0,088	-0,325	0,100	0,295	0,191	-0,425	0,337	0,096	0,675	-0,502	-0,421	0,081	0,084	-0,579	0,312	-0,170	0,081	-0,182	1,000
	**significance**	0,721	0,175	0,684	0,221	0,433	0,070	0,158	0,694	0,002	0,029	0,073	0,743	0,732	0,009	0,193	0,486	0,743	0,455	

Supplementary Table S4. Spearman's correlations in sepsis survivor's group, 2nd sample collected.

		Age	CircHist-B	CitrH3-B	PCA-B	Lactate-B	SOFATot-B	APTT-B	PCR-B	PCT-B	LEUCO-B	PMN-B	Plaq-B	Quick-B	DD-B	TropUS-B	FuncProtC	APACHEII	PROTOTAL	LAC6H
**Age**	**correlation coefficient**	1,000	0,269	0,665	0,143	-0,064	-0,017	1,000	0,286	0,395	-0,622	-0,473	-0,235	-0,657		-0,500	0,193	0,665	0,157	0,136
	**significance**		0,481	0,057	0,715	0,889	0,980	0,333	0,454	0,335	0,081	0,199	0,541	0,175		1,000	0,616	0,056	0,711	0,724
**CircHist-B**	**correlation coefficient**	0,269	1,000	0,504	-0,350	-0,114	0,000	0,500	0,200	-0,333	0,167	0,025	0,167	-0,543		-1,000	-0,267	-0,134	-0,048	-0,437
	**significance**	0,481		0,170	0,359	0,789	1,000	1,000	0,613	0,428	0,678	0,957	0,678	0,297		0,333	0,493	0,733	0,935	0,240
**CitrH3-B**	**correlation coefficient**	0,665	0,504	1,000	0,445	-0,039	0,313	1,000	-0,084	0,323	-0,244	0,072	-0,462	-0,486		-1,000	-0,076	0,398	0,349	-0,059
	**significance**	0,057	0,170		0,230	0,938	0,419	0,333	0,834	0,432	0,524	0,855	0,212	0,356		0,333	0,849	0,284	0,393	0,879
**PCA-B**	**correlation coefficient**	0,143	-0,350	0,445	1,000	0,051	0,380	0,500	-0,617	0,262	0,017	0,310	-0,533	0,257		0,500	0,383	0,269	0,167	-0,050
	**significance**	0,715	0,359	0,230		0,899	0,334	1,000	0,086	0,536	0,982	0,415	0,148	0,658		1,000	0,313	0,482	0,703	0,902
**Lactate-B**	**correlation coefficient**	-0,064	-0,114	-0,039	0,051	1,000	0,098	0,866	-0,342	0,185	0,038	-0,166	0,419	-0,224			-0,571	0,096	-0,296	0,520
	**significance**	0,889	0,789	0,938	0,899		0,821	0,667	0,412	0,714	0,930	0,696	0,311	1,000			0,149	0,811	0,531	0,198
**SOFATot-B**	**correlation coefficient**	-0,017	0,000	0,313	0,380	0,098	1,000	0,000	-0,069	0,200	0,552	-0,017	-0,449	-0,706		0,500	-0,242	0,470	0,701	-0,418
	**significance**	0,980	1,000	0,419	0,334	0,821		1,000	0,890	0,675	0,136	0,983	0,243	0,144		1,000	0,556	0,211	0,073	0,271
**APTT-B**	**correlation coefficient**	1,000	0,500	1,000	0,500	0,866	0,000	1,000	-0,500	1,000	-0,500	1,000	-0,500	-0,500			0,500	0,500	0,500	0,500
	**significance**	0,333	1,000	0,333	1,000	0,667	1,000		1,000	0,333	1,000	0,333	1,000	1,000			1,000	1,000	1,000	1,000
**PCR-B**	**correlation coefficient**	0,286	0,200	-0,084	-0,617	-0,342	-0,069	-0,500	1,000	0,476	-0,117	-0,452	0,383	-0,486		0,500	-0,350	0,479	-0,190	0,109
	**significance**	0,454	0,613	0,834	0,086	0,412	0,890	1,000		0,243	0,776	0,224	0,313	0,356		1,000	0,359	0,195	0,665	0,782
**PCT-B**	**correlation coefficient**	0,395	-0,333	0,323	0,262	0,185	0,200	1,000	0,476	1,000	-0,333	0,012	-0,119	-0,314		0,500	-0,286	0,747	0,000	0,611
	**significance**	0,335	0,428	0,432	0,536	0,714	0,675	0,333	0,243		0,428	0,990	0,793	0,564		1,000	0,501	0,040	1,000	0,118
**LEUCO-B**	**correlation coefficient**	-0,622	0,167	-0,244	0,017	0,038	0,552	-0,500	-0,117	-0,333	1,000	0,351	0,200	-0,257		0,500	-0,200	-0,185	0,024	-0,655
	**significance**	0,081	0,678	0,524	0,982	0,930	0,136	1,000	0,776	0,428		0,352	0,613	0,658		1,000	0,613	0,634	0,977	0,062
**PMN-B**	**correlation coefficient**	-0,473	0,025	0,072	0,310	-0,166	-0,017	1,000	-0,452	0,012	0,351	1,000	-0,134	0,290		0,500	0,159	-0,506	0,119	-0,152
	**significance**	0,199	0,957	0,855	0,415	0,696	0,983	0,333	0,224	0,990	0,352		0,731	0,600		1,000	0,683	0,166	0,793	0,692
**Plaq-B**	**correlation coefficient**	-0,235	0,167	-0,462	-0,533	0,419	-0,449	-0,500	0,383	-0,119	0,200	-0,134	1,000	0,086		-0,500	-0,383	-0,143	-0,810	0,210
	**significance**	0,541	0,678	0,212	0,148	0,311	0,243	1,000	0,313	0,793	0,613	0,731		0,919		1,000	0,313	0,714	0,022	0,586
**Quick-B**	**correlation coefficient**	-0,657	-0,543	-0,486	0,257	-0,224	-0,706	-0,500	-0,486	-0,314	-0,257	0,290	0,086	1,000		0,500	0,257	-0,667	-0,800	0,257
	**significance**	0,175	0,297	0,356	0,658	1,000	0,144	1,000	0,356	0,564	0,658	0,600	0,919			1,000	0,658	0,161	0,133	0,658
**DD-B**	**correlation coefficient**																			
	**significance**																			
**TropUS-B**	**correlation coefficient**	-0,500	-1,000	-1,000	0,500		0,500		0,500	0,500	0,500	0,500	-0,500	0,500		1,000	1,000	0,500	0,500	-1,000
	**significance**	1,000	0,333	0,333	1,000		1,000		1,000	1,000	1,000	1,000	1,000	1,000			0,333	1,000	1,000	0,333
**FuncProtC**	**correlation coefficient**	0,193	-0,267	-0,076	0,383	-0,571	-0,242	0,500	-0,350	-0,286	-0,200	0,159	-0,383	0,257		1,000	1,000	-0,101	0,190	-0,328
	**significance**	0,616	0,493	0,849	0,313	0,149	0,556	1,000	0,359	0,501	0,613	0,683	0,313	0,658		0,333		0,800	0,665	0,386
**APACHEII**	**correlation coefficient**	0,665	-0,134	0,398	0,269	0,096	0,470	0,500	0,479	0,747	-0,185	-0,506	-0,143	-0,667		0,500	-0,101	1,000	0,024	0,144
	**significance**	0,056	0,733	0,284	0,482	0,811	0,211	1,000	0,195	0,040	0,634	0,166	0,714	0,161		1,000	0,800		0,968	0,710
**PROTOTAL**	**correlation coefficient**	0,157	-0,048	0,349	0,167	-0,296	0,701	0,500	-0,190	0,000	0,024	0,119	-0,810	-0,800		0,500	0,190	0,024	1,000	-0,347
	**significance**	0,711	0,935	0,393	0,703	0,531	0,073	1,000	0,665	1,000	0,977	0,793	0,022	0,133		1,000	0,665	0,968		0,400
**LAC6H**	**correlation coefficient**	0,136	-0,437	-0,059	-0,050	0,520	-0,418	0,500	0,109	0,611	-0,655	-0,152	0,210	0,257		-1,000	-0,328	0,144	-0,347	1,000
	**significance**	0,724	0,240	0,879	0,902	0,198	0,271	1,000	0,782	0,118	0,062	0,692	0,586	0,658		0,333	0,386	0,710	0,400	

Supplementary Table S5. Spearman's in sepsis survivor’s group, 3rd sample collected.

		Age	CircHist-C	CitrH3-C	PCA-C	Lactate-C	SOFATot-C	APTT-C	PCR-C	PCT-C	LEUCO-C	PMN-C	Plaq-C	Quick-C	DD-C	TropUS-C	FuncProtC	APACHEII	PROTOTAL	LAC6H
Age	correlation coefficient	1,000	0,563	0,173	-0,059	-0,206	0,086	0,500	-0,143	-0,400	-0,200	0,609	-0,600	-0,400			0,193	0,665	0,157	0,136
	significance		0,121	0,652	0,888	0,711	0,919	1,000	0,803	0,517	0,714	0,211	0,242	0,750			0,616	0,056	0,711	0,724
CircHist-C	correlation coefficient	0,563	1,000	0,418	-0,017	0,029	0,771	-0,500	-0,086	0,200	0,029	-0,029	-0,257	-0,800			0,000	0,437	-0,524	0,580
	significance	0,121		0,262	0,982	0,978	0,103	1,000	0,919	0,783	1,000	0,983	0,658	0,333			1,000	0,241	0,197	0,108
CitrH3-C	correlation coefficient	0,173	0,418	1,000	-0,259	0,324	0,486	-0,500	-0,143	0,200	-0,143	0,232	-0,543	-0,800			-0,184	0,030	0,192	0,283
	significance	0,652	0,262		0,497	0,556	0,356	1,000	0,803	0,783	0,803	0,650	0,297	0,333			0,633	0,943	0,647	0,453
PCA-C	correlation coefficient	-0,059	-0,017	-0,259	1,000	0,029	0,657	1,000	-0,714	-0,700	-0,543	0,029	-0,371	0,400			0,667	0,067	0,000	-0,412
	significance	0,888	0,982	0,497		0,978	0,175	0,333	0,136	0,233	0,297	0,983	0,497	0,750			0,059	0,868	1,000	0,271
Lactate-C	correlation coefficient	-0,206	0,029	0,324	0,029	1,000	0,029	-0,500	-0,647	0,000	-0,618	-0,015	-0,500	-0,316			0,618	-0,851	0,462	-0,015
	significance	0,711	0,978	0,556	0,978		0,978	1,000	0,178	1,000	0,200	0,967	0,300	1,000			0,200	0,044	0,433	0,967
SOFATot-C	correlation coefficient	0,086	0,771	0,486	0,657	0,029	1,000	0,500	-0,543	-0,600	-0,200	-0,145	-0,371	-0,400			0,200	0,464	-0,100	-0,029
	significance	0,919	0,103	0,356	0,175	0,978		1,000	0,297	0,350	0,714	0,778	0,497	0,750			0,714	0,372	0,950	0,983
APTT-C	correlation coefficient	0,500	-0,500	-0,500	1,000	-0,500	0,500	1,000	-0,500	-0,500	-0,500	1,000	-0,500	1,000			0,500	0,866	0,500	-0,500
	significance	1,000	1,000	1,000	0,333	1,000	1,000		1,000	1,000	1,000	0,333	1,000	0,333			1,000	0,667	1,000	1,000
PCR-C	correlation coefficient	-0,143	-0,086	-0,143	-0,714	-0,647	-0,543	-0,500	1,000	0,700	0,714	0,029	0,657	0,400			-0,714	0,261	-0,600	0,348
	significance	0,803	0,919	0,803	0,136	0,178	0,297	1,000		0,233	0,136	0,983	0,175	0,750			0,136	0,617	0,350	0,494
PCT-C	correlation coefficient	-0,400	0,200	0,200	-0,700	0,000	-0,600	-0,500	0,700	1,000	0,200	0,300	0,300	0,400			-0,200	-0,359	-1,000	0,700
	significance	0,517	0,783	0,783	0,233	1,000	0,350	1,000	0,233		0,783	0,683	0,683	0,750			0,783	0,633	0,083	0,233
LEUCO-C	correlation coefficient	-0,200	0,029	-0,143	-0,543	-0,618	-0,200	-0,500	0,714	0,200	1,000	-0,580	0,829	-0,200			-1,000	0,377	-0,500	0,232
	significance	0,714	1,000	0,803	0,297	0,200	0,714	1,000	0,136	0,783		0,244	0,058	0,917			0,003	0,472	0,450	0,650
PMN-C	correlation coefficient	0,609	-0,029	0,232	0,029	-0,015	-0,145	1,000	0,029	0,300	-0,580	1,000	-0,667	0,400			0,580	0,074	0,154	-0,397
	significance	0,211	0,983	0,650	0,983	0,967	0,778	0,333	0,983	0,683	0,244		0,161	0,750			0,244	0,886	0,833	0,428
Plaq-C	correlation coefficient	-0,600	-0,257	-0,543	-0,371	-0,500	-0,371	-0,500	0,657	0,300	0,829	-0,667	1,000	0,400			-0,829	0,116	-0,500	0,580
	significance	0,242	0,658	0,297	0,497	0,300	0,497	1,000	0,175	0,683	0,058	0,161		0,750			0,058	0,839	0,450	0,244
Quick-C	correlation coefficient	-0,400	-0,800	-0,800	0,400	-0,316	-0,400	1,000	0,400	0,400	-0,200	0,400	0,400	1,000			0,200	-0,316	0,500	0,400
	significance	0,750	0,333	0,333	0,750	1,000	0,750	0,333	0,750	0,750	0,917	0,750	0,750				0,917	1,000	1,000	0,750
DD-C	correlation coefficient																			
	significance																			
TropUS-C	correlation coefficient																			
	significance																			
FuncProtC	correlation coefficient	0,193	0,000	-0,184	0,667	0,618	0,200	0,500	-0,714	-0,200	-1,000	0,580	-0,829	0,200			1,000	-0,101	0,190	-0,328
	significance	0,616	1,000	0,633	0,059	0,200	0,714	1,000	0,136	0,783	0,003	0,244	0,058	0,917				0,800	0,665	0,386
APACHEII	correlation coefficient	0,665	0,437	0,030	0,067	-0,851	0,464	0,866	0,261	-0,359	0,377	0,074	0,116	-0,316			-0,101	1,000	0,024	0,144
	significance	0,056	0,241	0,943	0,868	0,044	0,372	0,667	0,617	0,633	0,472	0,886	0,839	1,000			0,800		0,968	0,710
PROTOTAL	correlation coefficient	0,157	-0,524	0,192	0,000	0,462	-0,100	0,500	-0,600	-1,000	-0,500	0,154	-0,500	0,500			0,190	0,024	1,000	-0,347
	significance	0,711	0,197	0,647	1,000	0,433	0,950	1,000	0,350	0,083	0,450	0,833	0,450	1,000			0,665	0,968		0,400
LAC6H	correlation coefficient	0,136	0,580	0,283	-0,412	-0,015	-0,029	-0,500	0,348	0,700	0,232	-0,397	0,580	0,400			-0,328	0,144	-0,347	1,000
	significance	0,724	0,108	0,453	0,271	0,967	0,983	1,000	0,494	0,233	0,650	0,428	0,244	0,750			0,386	0,710	0,400	

Supplementary Table S6. Spearman's correlations in SS survivor's group, 1st sample collected.

		Age	CircHist-A	CitrH3-A	PCA-A	Lactate-A	SOFATot-A	APTT-A	PCR-A	PCT-A	LEUCO-A	PMN-A	Plaq-A	Quick-A	DD-A	TropUS-A	FuncProtC	APACHEII	PROTOTAL	LAC6H
**Age**	**correlation coefficient**	1,000	0,133	-0,133	-0,025	-0,646	0,191	-0,275	-0,249	0,456	0,246	0,693	0,114	-0,072	0,379	-0,144	-0,163	0,651	0,505	-0,604
	**significance**		0,586	0,586	0,920	0,003	0,433	0,254	0,304	0,050	0,311	0,001	0,642	0,770	0,110	0,557	0,505	0,003	0,027	0,006
**CircHist-A**	**correlation coefficient**	0,133	1,000	-0,205	-0,632	-0,465	0,456	0,600	-0,505	-0,256	-0,153	0,107	-0,561	-0,777	0,661	0,716	-0,726	0,047	0,498	0,037
	**significance**	0,586		0,399	0,004	0,045	0,050	0,007	0,027	0,290	0,533	0,663	0,012	0,000	0,002	0,001	0,000	0,847	0,030	0,881
**CitrH3-A**	**correlation coefficient**	-0,133	-0,205	1,000	0,370	0,486	0,156	-0,258	-0,016	-0,302	0,339	-0,016	0,453	0,095	-0,312	-0,354	0,446	0,309	-0,151	0,172
	**significance**	0,586	0,399		0,119	0,035	0,523	0,286	0,949	0,209	0,156	0,949	0,052	0,700	0,193	0,137	0,056	0,198	0,538	0,482
**PCA-A**	**correlation coefficient**	-0,025	-0,632	0,370	1,000	0,400	-0,679	-0,651	0,567	0,419	0,667	0,091	0,933	0,861	-0,702	-0,677	0,951	-0,114	-0,267	0,168
	**significance**	0,920	0,004	0,119		0,090	0,001	0,003	0,011	0,074	0,002	0,710	0,000	0,000	0,001	0,001	0,000	0,642	0,270	0,491
**Lactate-A**	**correlation coefficient**	-0,646	-0,465	0,486	0,400	1,000	-0,072	-0,089	0,565	-0,242	-0,077	-0,284	0,282	0,451	-0,505	-0,156	0,465	-0,325	-0,516	0,574
	**significance**	0,003	0,045	0,035	0,090		0,770	0,716	0,012	0,318	0,753	0,238	0,241	0,053	0,027	0,523	0,045	0,175	0,024	0,010
**SOFATot-A**	**correlation coefficient**	0,191	0,456	0,156	-0,679	-0,072	1,000	0,270	-0,518	-0,477	-0,532	0,305	-0,605	-0,700	0,642	0,442	-0,656	0,561	0,468	-0,240
	**significance**	0,433	0,050	0,523	0,001	0,770		0,263	0,023	0,039	0,019	0,204	0,006	0,001	0,003	0,058	0,002	0,012	0,043	0,322
**APTT-A**	**correlation coefficient**	-0,275	0,600	-0,258	-0,651	-0,089	0,270	1,000	-0,205	-0,251	-0,351	-0,363	-0,714	-0,602	0,365	0,828	-0,609	-0,446	-0,046	0,525
	**significance**	0,254	0,007	0,286	0,003	0,716	0,263		0,399	0,300	0,141	0,126	0,001	0,006	0,124	0,000	0,006	0,056	0,853	0,021
**PCR-A**	**correlation coefficient**	-0,249	-0,505	-0,016	0,567	0,565	-0,518	-0,205	1,000	0,335	0,056	0,014	0,402	0,818	-0,605	-0,263	0,479	-0,442	-0,574	0,465
	**significance**	0,304	0,027	0,949	0,011	0,012	0,023	0,399		0,161	0,819	0,955	0,088	0,000	0,006	0,276	0,038	0,058	0,010	0,045
**PCT-A**	**correlation coefficient**	0,456	-0,256	-0,302	0,419	-0,242	-0,477	-0,251	0,335	1,000	0,453	0,365	0,428	0,482	-0,049	-0,202	0,328	-0,123	0,012	-0,130
	**significance**	0,050	0,290	0,209	0,074	0,318	0,039	0,300	0,161		0,052	0,124	0,067	0,036	0,842	0,408	0,170	0,616	0,960	0,596
**LEUCO-A**	**correlation coefficient**	0,246	-0,153	0,339	0,667	-0,077	-0,532	-0,351	0,056	0,453	1,000	0,072	0,767	0,370	-0,216	-0,435	0,656	-0,016	0,019	0,025
	**significance**	0,311	0,533	0,156	0,002	0,753	0,019	0,141	0,819	0,052		0,770	0,000	0,119	0,375	0,063	0,002	0,949	0,937	0,920
**PMN-A**	**correlation coefficient**	0,693	0,107	-0,016	0,091	-0,284	0,305	-0,363	0,014	0,365	0,072	1,000	0,079	0,039	0,270	-0,011	-0,058	0,612	0,491	-0,516
	**significance**	0,001	0,663	0,949	0,710	0,238	0,204	0,126	0,955	0,124	0,770		0,748	0,875	0,263	0,966	0,814	0,005	0,033	0,024
**Plaq-A**	**correlation coefficient**	0,114	-0,561	0,453	0,933	0,282	-0,605	-0,714	0,402	0,428	0,767	0,079	1,000	0,765	-0,602	-0,777	0,896	0,046	-0,244	0,042
	**significance**	0,642	0,012	0,052	0,000	0,241	0,006	0,001	0,088	0,067	0,000	0,748		0,000	0,006	0,000	0,000	0,853	0,314	0,864
**QuickA**	**correlation coefficient**	-0,072	-0,777	0,095	0,861	0,451	-0,700	-0,602	0,818	0,482	0,370	0,039	0,765	1,000	-0,746	-0,658	0,804	-0,219	-0,484	0,198
	**significance**	0,770	0,000	0,700	0,000	0,053	0,001	0,006	0,000	0,036	0,119	0,875	0,000		0,000	0,002	0,000	0,367	0,036	0,416
**DD-A**	**correlation coefficient**	0,379	0,661	-0,312	-0,702	-0,505	0,642	0,365	-0,605	-0,049	-0,216	0,270	-0,602	-0,746	1,000	0,612	-0,756	0,279	0,714	-0,388
	**significance**	0,110	0,002	0,193	0,001	0,027	0,003	0,124	0,006	0,842	0,375	0,263	0,006	0,000		0,005	0,000	0,247	0,001	0,101
**TropUS-A**	**correlation coefficient**	-0,144	0,716	-0,354	-0,677	-0,156	0,442	0,828	-0,263	-0,202	-0,435	-0,011	-0,777	-0,658	0,612	1,000	-0,723	-0,261	0,298	0,274
	**significance**	0,557	0,001	0,137	0,001	0,523	0,058	0,000	0,276	0,408	0,063	0,966	0,000	0,002	0,005		0,000	0,280	0,215	0,257
**FuncProtC**	**correlation coefficient**	-0,163	-0,726	0,446	0,951	0,465	-0,656	-0,609	0,479	0,328	0,656	-0,058	0,896	0,804	-0,756	-0,723	1,000	-0,146	-0,368	0,177
	**significance**	0,505	0,000	0,056	0,000	0,045	0,002	0,006	0,038	0,170	0,002	0,814	0,000	0,000	0,000	0,000		0,552	0,121	0,468
**APACHEII**	**correlation coefficient**	0,651	0,047	0,309	-0,114	-0,325	0,561	-0,446	-0,442	-0,123	-0,016	0,612	0,046	-0,219	0,279	-0,261	-0,146	1,000	0,477	-0,635
	**significance**	0,003	0,847	0,198	0,642	0,175	0,012	0,056	0,058	0,616	0,949	0,005	0,853	0,367	0,247	0,280	0,552		0,039	0,003
**PROTOTAL**	**correlation coefficient**	0,505	0,498	-0,151	-0,267	-0,516	0,468	-0,046	-0,574	0,012	0,019	0,491	-0,244	-0,484	0,714	0,298	-0,368	0,477	1,000	-0,495
	**significance**	0,027	0,030	0,538	0,270	0,024	0,043	0,853	0,010	0,960	0,937	0,033	0,314	0,036	0,001	0,215	0,121	0,039		0,031
**LAC6H**	**correlation coefficient**	-0,604	0,037	0,172	0,168	0,574	-0,240	0,525	0,465	-0,130	0,025	-0,516	0,042	0,198	-0,388	0,274	0,177	-0,635	-0,495	1,000
	**significance**	0,006	0,881	0,482	0,491	0,010	0,322	0,021	0,045	0,596	0,920	0,024	0,864	0,416	0,101	0,257	0,468	0,003	0,031	

Supplementary Table S7. Spearman's correlations in SS survivor's group, 2nd sample collected.

		Age	CircHist-B	CitrH3-B	PCA-B	Lactate-B	SOFATot-B	APTT-B	PCR-B	PCT-B	LEUCO-B	PMN-B	Plaq-B	Quick-B	DD-B	TropUS-B	FuncProtC	APACHEII	PROTOTAL	LAC6H
**Age**	**correlation coefficient**	1,000	-0,410	-0,277	-0,203	-0,291	-0,198	-0,588	-0,037	-0,166	0,161	0,410	-0,147	0,503	0,500	-0,205	0,102	0,527	0,167	0,058
	**significance**		0,210	0,408	0,548	0,409	0,579	0,233	0,918	0,621	0,634	0,209	0,665	0,116	1,000	0,767	0,764	0,098	0,620	0,874
**CircHist-B**	**correlation coefficient**	-0,410	1,000	0,464	-0,091	0,347	0,459	-0,771	0,318	0,137	-0,382	0,023	-0,055	-0,296	-0,500	0,900	-0,556	-0,560	-0,247	-0,340
	**significance**	0,210		0,155	0,796	0,324	0,183	0,103	0,342	0,687	0,248	0,951	0,881	0,374	1,000	0,083	0,079	0,077	0,462	0,334
**CitrH3-B**	**correlation coefficient**	-0,277	0,464	1,000	0,564	-0,097	0,563	-0,657	0,355	0,374	-0,273	0,239	-0,100	0,246	-0,500	0,600	-0,114	-0,155	-0,511	0,201
	**significance**	0,408	0,155		0,076	0,792	0,095	0,175	0,286	0,256	0,418	0,475	0,776	0,462	1,000	0,350	0,739	0,647	0,111	0,576
**PCA-B**	**correlation coefficient**	-0,203	-0,091	0,564	1,000	-0,395	-0,092	0,086	-0,027	0,487	-0,227	0,152	0,155	0,528	-0,500	-0,900	0,606	0,214	-0,338	0,347
	**significance**	0,548	0,796	0,076		0,257	0,802	0,919	0,946	0,131	0,503	0,655	0,654	0,098	1,000	0,083	0,052	0,525	0,307	0,324
**Lactate-B**	**correlation coefficient**	-0,291	0,347	-0,097	-0,395	1,000	0,650	0,543	0,450	-0,165	0,158	0,157	-0,328	-0,787	1,000	0,600	-0,571	-0,177	0,532	-0,251
	**significance**	0,409	0,324	0,792	0,257		0,065	0,297	0,194	0,646	0,663	0,660	0,353	0,009	0,333	0,350	0,089	0,622	0,116	0,512
**SOFATot-B**	**correlation coefficient**	-0,198	0,459	0,563	-0,092	0,650	1,000	0,316	0,404	-0,242	-0,220	0,180	-0,361	-0,365	1,000	0,872	-0,740	-0,074	0,040	-0,110
	**significance**	0,579	0,183	0,095	0,802	0,065		0,600	0,247	0,494	0,538	0,619	0,303	0,295	0,333	0,100	0,018	0,839	0,914	0,759
**APTT-B**	**correlation coefficient**	-0,588	-0,771	-0,657	0,086	0,543	0,316	1,000	-0,371	-0,377	0,086	-0,143	-0,257	-0,600	1,000	-0,400	0,029	0,522	0,771	-0,100
	**significance**	0,233	0,103	0,175	0,919	0,297	0,600		0,497	0,472	0,919	0,803	0,658	0,242	0,333	0,750	1,000	0,300	0,103	0,950
**PCR-B**	**correlation coefficient**	-0,037	0,318	0,355	-0,027	0,450	0,404	-0,371	1,000	0,433	0,209	0,547	0,118	0,050	0,500	0,900	-0,005	-0,068	-0,242	0,213
	**significance**	0,918	0,342	0,286	0,946	0,194	0,247	0,497		0,184	0,539	0,086	0,735	0,887	1,000	0,083	0,995	0,844	0,470	0,552
**PCT-B**	**correlation coefficient**	-0,166	0,137	0,374	0,487	-0,165	-0,242	-0,377	0,433	1,000	0,027	-0,088	0,569	0,311	-0,500	0,051	0,475	-0,164	-0,451	0,381
	**significance**	0,621	0,687	0,256	0,131	0,646	0,494	0,472	0,184		0,940	0,797	0,071	0,349	1,000	1,000	0,141	0,626	0,164	0,274
**LEUCO-B**	**correlation coefficient**	0,161	-0,382	-0,273	-0,227	0,158	-0,220	0,086	0,209	0,027	1,000	0,248	-0,055	-0,032	1,000	0,300	0,287	-0,169	0,005	0,766
	**significance**	0,634	0,248	0,418	0,503	0,663	0,538	0,919	0,539	0,940		0,459	0,881	0,930	0,333	0,683	0,390	0,619	0,994	0,013
**PMN-B**	**correlation coefficient**	0,410	0,023	0,239	0,152	0,157	0,180	-0,143	0,547	-0,088	0,248	1,000	-0,336	0,270	1,000	-0,200	0,154	0,350	0,134	0,160
	**significance**	0,209	0,951	0,475	0,655	0,660	0,619	0,803	0,086	0,797	0,459		0,311	0,417	0,333	0,783	0,646	0,288	0,691	0,658
**Plaq-B**	**correlation coefficient**	-0,147	-0,055	-0,100	0,155	-0,328	-0,361	-0,257	0,118	0,569	-0,055	-0,336	1,000	0,364	-1,000	0,000	0,446	-0,415	-0,452	-0,012
	**significance**	0,665	0,881	0,776	0,654	0,353	0,303	0,658	0,735	0,071	0,881	0,311		0,268	0,333	1,000	0,169	0,205	0,164	0,980
**Quick-B**	**correlation coefficient**	0,503	-0,296	0,246	0,528	-0,787	-0,365	-0,600	0,050	0,311	-0,032	0,270	0,364	1,000	-0,500	-0,800	0,580	0,233	-0,597	0,442
	**significance**	0,116	0,374	0,462	0,098	0,009	0,295	0,242	0,887	0,349	0,930	0,417	0,268		1,000	0,133	0,065	0,486	0,056	0,199
**DD-B**	**correlation coefficient**	0,500	-0,500	-0,500	-0,500	1,000	1,000	1,000	0,500	-0,500	1,000	1,000	-1,000	-0,500	1,000		-0,500	0,500	1,000	-0,500
	**significance**	1,000	1,000	1,000	1,000	0,333	0,333	0,333	1,000	1,000	0,333	0,333	0,333	1,000			1,000	1,000	0,333	1,000
**TropUS-B**	**correlation coefficient**	-0,205	0,900	0,600	-0,900	0,600	0,872	-0,400	0,900	0,051	0,300	-0,200	0,000	-0,800		1,000	-1,000	-0,900	-0,200	0,000
	**significance**	0,767	0,083	0,350	0,083	0,350	0,100	0,750	0,083	1,000	0,683	0,783	1,000	0,133			0,017	0,083	0,783	1,000
**FuncProtC**	**correlation coefficient**	0,102	-0,556	-0,114	0,606	-0,571	-0,740	0,029	-0,005	0,475	0,287	0,154	0,446	0,580	-0,500	-1,000	1,000	0,260	-0,201	0,432
	**significance**	0,764	0,079	0,739	0,052	0,089	0,018	1,000	0,995	0,141	0,390	0,646	0,169	0,065	1,000	0,017		0,435	0,548	0,213
**APACHEII**	**correlation coefficient**	0,527	-0,560	-0,155	0,214	-0,177	-0,074	0,522	-0,068	-0,164	-0,169	0,350	-0,415	0,233	0,500	-0,900	0,260	1,000	0,451	-0,085
	**significance**	0,098	0,077	0,647	0,525	0,622	0,839	0,300	0,844	0,626	0,619	0,288	0,205	0,486	1,000	0,083	0,435		0,164	0,815
**PROTOTAL**	**correlation coefficient**	0,167	-0,247	-0,511	-0,338	0,532	0,040	0,771	-0,242	-0,451	0,005	0,134	-0,452	-0,597	1,000	-0,200	-0,201	0,451	1,000	-0,541
	**significance**	0,620	0,462	0,111	0,307	0,116	0,914	0,103	0,470	0,164	0,994	0,691	0,164	0,056	0,333	0,783	0,548	0,164		0,109
**LAC6H**	**correlation coefficient**	0,058	-0,340	0,201	0,347	-0,251	-0,110	-0,100	0,213	0,381	0,766	0,160	-0,012	0,442	-0,500	0,000	0,432	-0,085	-0,541	1,000
	**significance**	0,874	0,334	0,576	0,324	0,512	0,759	0,950	0,552	0,274	0,013	0,658	0,980	0,199	1,000	1,000	0,213	0,815	0,109	

Supplementary Table S8. Spearman's correlations in SS survivor’s group, 3rd sample collected.

		Age	CircHist-C	CitrH3-C	PCA-C	Lactate-C	SOFATot-C	APTT-C	PCR-C	PCT-C	LEUCO-C	PMN-C	Plaq-C	Quick-C	DD-C	TropUS-C	FuncProtC	APACHEII	PROTOTAL	LAC6H
**Age**	**correlation coefficient**	1,000	0,092	-0,194	-0,309	-0,513	-0,664	-0,791	0,198	-0,179	-0,049	-0,031	0,463	-0,180	0,000	0,500	0,102	0,527	0,167	0,058
	**significance**		0,789	0,563	0,353	0,159	0,056	0,133	0,582	0,619	0,897	0,938	0,180	0,635	1,000	0,450	0,764	0,098	0,620	0,874
**CircHist-C**	**correlation coefficient**	0,092	1,000	-0,073	0,282	0,234	0,555	-0,400	0,745	0,624	0,758	0,539	0,236	0,335	0,000	-0,700	-0,009	0,205	-0,352	0,450
	**significance**	0,789		0,833	0,402	0,540	0,126	0,517	0,017	0,060	0,015	0,114	0,513	0,375	1,000	0,233	0,983	0,543	0,287	0,194
**CitrH3-C**	**correlation coefficient**	-0,194	-0,073	1,000	0,128	-0,471	-0,262	-0,051	0,389	0,182	-0,225	-0,486	0,419	-0,109	-1,000	-0,300	0,292	-0,486	-0,611	0,122
	**significance**	0,563	0,833		0,708	0,202	0,489	1,000	0,266	0,614	0,529	0,157	0,227	0,780	0,083	0,683	0,379	0,130	0,049	0,736
**PCA-C**	**correlation coefficient**	-0,309	0,282	0,128	1,000	-0,176	0,143	-0,300	0,164	0,115	0,467	0,115	0,273	0,628	-0,600	-1,000	0,574	0,055	-0,397	0,790
	**significance**	0,353	0,402	0,708		0,653	0,713	0,683	0,657	0,759	0,179	0,759	0,448	0,077	0,417	0,017	0,069	0,875	0,226	0,009
**Lactate-C**	**correlation coefficient**	-0,513	0,234	-0,471	-0,176	1,000	0,741	0,200	0,184	0,100	0,544	0,552	-0,444	-0,192	1,000	0,400	-0,444	-0,252	0,515	-0,331
	**significance**	0,159	0,540	0,202	0,653		0,041	0,783	0,634	0,800	0,135	0,128	0,234	0,646	0,333	0,750	0,234	0,506	0,158	0,415
**SOFATot-C**	**correlation coefficient**	-0,664	0,555	-0,262	0,143	0,741	1,000	-0,600	0,134	0,504	0,429	0,588	-0,563	0,235	0,800	0,205	-0,563	-0,397	-0,059	0,030
	**significance**	0,056	0,126	0,489	0,713	0,041		0,350	0,732	0,170	0,250	0,102	0,120	0,565	0,333	0,733	0,120	0,286	0,881	0,943
**APTT-C**	**correlation coefficient**	-0,791	-0,400	-0,051	-0,300	0,200	-0,600	1,000	-0,300	-0,400	-0,100	-0,300	0,600	0,000	-0,500	-1,000	0,000	0,103	0,200	-0,600
	**significance**	0,133	0,517	1,000	0,683	0,783	0,350		0,683	0,517	0,950	0,683	0,350	1,000	1,000	0,333	1,000	0,900	0,783	0,350
**PCR-C**	**correlation coefficient**	0,198	0,745	0,389	0,164	0,184	0,134	-0,300	1,000	0,418	0,697	0,442	0,552	0,084	0,000	-0,700	0,480	0,146	-0,274	0,285
	**significance**	0,582	0,017	0,266	0,657	0,634	0,732	0,683		0,233	0,031	0,204	0,105	0,834	1,000	0,233	0,162	0,687	0,440	0,454
**PCT-C**	**correlation coefficient**	-0,179	0,624	0,182	0,115	0,100	0,504	-0,400	0,418	1,000	0,382	0,527	-0,261	0,494	0,200	0,300	0,085	0,036	-0,354	0,100
	**significance**	0,619	0,060	0,614	0,759	0,800	0,170	0,517	0,233		0,279	0,123	0,470	0,180	0,917	0,683	0,818	0,926	0,314	0,800
**LEUCO-C**	**correlation coefficient**	-0,049	0,758	-0,225	0,467	0,544	0,429	-0,100	0,697	0,382	1,000	0,661	0,152	0,218	0,800	-0,300	0,413	0,310	0,110	0,477
	**significance**	0,897	0,015	0,529	0,179	0,135	0,250	0,950	0,031	0,279		0,044	0,682	0,572	0,333	0,683	0,235	0,380	0,764	0,198
**PMN-C**	**correlation coefficient**	-0,031	0,539	-0,486	0,115	0,552	0,588	-0,300	0,442	0,527	0,661	1,000	-0,152	0,126	1,000	0,300	0,164	0,085	0,018	0,134
	**significance**	0,938	0,114	0,157	0,759	0,128	0,102	0,683	0,204	0,123	0,044		0,682	0,751	0,083	0,683	0,649	0,818	0,966	0,732
**Plaq-C**	**correlation coefficient**	0,463	0,236	0,419	0,273	-0,444	-0,563	0,600	0,552	-0,261	0,152	-0,152	1,000	0,008	-0,800	-0,300	0,723	0,267	-0,378	0,301
	**significance**	0,180	0,513	0,227	0,448	0,234	0,120	0,350	0,105	0,470	0,682	0,682		0,992	0,333	0,683	0,022	0,452	0,280	0,428
**Quick-C**	**correlation coefficient**	-0,180	0,335	-0,109	0,628	-0,192	0,235	0,000	0,084	0,494	0,218	0,126	0,008	1,000	-0,400	-0,400	0,345	0,546	-0,252	0,204
	**significance**	0,635	0,375	0,780	0,077	0,646	0,565	1,000	0,834	0,180	0,572	0,751	0,992		0,750	0,750	0,360	0,131	0,507	0,631
**DD-C**	**correlation coefficient**	0,000	0,000	-1,000	-0,600	1,000	0,800	-0,500	0,000	0,200	0,800	1,000	-0,800	-0,400	1,000	0,500	-0,600	0,400	1,000	-0,600
	**significance**	1,000	1,000	0,083	0,417	0,333	0,333	1,000	1,000	0,917	0,333	0,083	0,333	0,750		1,000	0,417	0,750	0,083	0,417
**TropUS-C**	**correlation coefficient**	0,500	-0,700	-0,300	-1,000	0,400	0,205	-1,000	-0,700	0,300	-0,300	0,300	-0,300	-0,400	0,500	1,000	-0,900	-0,100	0,359	-0,872
	**significance**	0,450	0,233	0,683	0,017	0,750	0,733	0,333	0,233	0,683	0,683	0,683	0,683	0,750	1,000		0,083	0,950	0,633	0,067
**FuncProtC**	**correlation coefficient**	0,102	-0,009	0,292	0,574	-0,444	-0,563	0,000	0,480	0,085	0,413	0,164	0,723	0,345	-0,600	-0,900	1,000	0,260	-0,201	0,432
	**significance**	0,764	0,983	0,379	0,069	0,234	0,120	1,000	0,162	0,818	0,235	0,649	0,022	0,360	0,417	0,083		0,435	0,548	0,213
**APACHEII**	**correlation coefficient**	0,527	0,205	-0,486	0,055	-0,252	-0,397	0,103	0,146	0,036	0,310	0,085	0,267	0,546	0,400	-0,100	0,260	1,000	0,451	-0,085
	**significance**	0,098	0,543	0,130	0,875	0,506	0,286	0,900	0,687	0,926	0,380	0,818	0,452	0,131	0,750	0,950	0,435		0,164	0,815
**PROTOTAL**	**correlation coefficient**	0,167	-0,352	-0,611	-0,397	0,515	-0,059	0,200	-0,274	-0,354	0,110	0,018	-0,378	-0,252	1,000	0,359	-0,201	0,451	1,000	-0,541
	**significance**	0,620	0,287	0,049	0,226	0,158	0,881	0,783	0,440	0,314	0,764	0,966	0,280	0,507	0,083	0,633	0,548	0,164		0,109
**LAC6H**	**correlation coefficient**	0,058	0,450	0,122	0,790	-0,331	0,030	-0,600	0,285	0,100	0,477	0,134	0,301	0,204	-0,600	-0,872	0,432	-0,085	-0,541	1,000
	**significance**	0,874	0,194	0,736	0,009	0,415	0,943	0,350	0,454	0,800	0,198	0,732	0,428	0,631	0,417	0,067	0,213	0,815	0,109	

Supplementary Table S9. Spearman's correlations in SS non-survivors' group, 1st sample collected.

		Age	CircHist-A	CitrH3-A	PCA-A	Lactate-A	SOFATot-A	APTT-A	PCR-A	PCT-A	LEUCO-A	PMN-A	Plaq-A	Quick-A	DD-A	TropUS-A	FuncProtC	APACHEII	PROTOTAL	LAC6H
**Age**	**correlation coefficient**	1,000	-0,865	-0,954	0,363	-0,617	0,131	0,291	0,090	0,140	-0,561	0,452	0,159	0,371	-0,165	0,343	0,284	0,284	-0,054	-0,342
	**significance**		0,000	0,000	0,127	0,005	0,593	0,227	0,713	0,569	0,012	0,052	0,516	0,117	0,499	0,150	0,239	0,239	0,827	0,152
**CircHist-A**	**correlation coefficient**	-0,865	1,000	0,914	-0,117	0,851	-0,124	0,036	-0,264	-0,257	0,803	-0,619	-0,161	-0,614	-0,007	-0,069	-0,284	-0,284	-0,160	0,542
	**significance**	0,000		0,000	0,634	0,000	0,614	0,884	0,274	0,288	0,000	0,005	0,511	0,005	0,977	0,778	0,239	0,239	0,513	0,017
**CitrH3-A**	**correlation coefficient**	-0,954	0,914	1,000	-0,292	0,696	-0,157	-0,205	-0,199	-0,241	0,629	-0,572	-0,198	-0,489	0,065	-0,199	-0,317	-0,317	-0,024	0,473
	**significance**	0,000	0,000		0,225	0,001	0,521	0,401	0,413	0,319	0,004	0,011	0,418	0,034	0,791	0,413	0,186	0,186	0,923	0,041
**PCA-A**	**correlation coefficient**	0,363	-0,117	-0,292	1,000	0,078	0,798	0,724	0,471	0,614	0,147	0,201	0,791	0,008	0,418	0,233	0,824	0,824	0,344	-0,148
	**significance**	0,127	0,634	0,225		0,751	0,000	0,000	0,042	0,005	0,549	0,409	0,000	0,974	0,075	0,338	0,000	0,000	0,150	0,544
**Lactate-A**	**correlation coefficient**	-0,617	0,851	0,696	0,078	1,000	-0,159	0,401	-0,464	-0,377	0,986	-0,752	-0,196	-0,874	-0,163	0,298	-0,259	-0,259	-0,444	0,758
	**significance**	0,005	0,000	0,001	0,751		0,516	0,089	0,045	0,112	0,000	0,000	0,422	0,000	0,504	0,216	0,284	0,284	0,057	0,000
**SOFATot-A**	**correlation coefficient**	0,131	-0,124	-0,157	0,798	-0,159	1,000	0,322	0,816	0,882	-0,124	0,468	0,995	0,377	0,785	-0,248	0,968	0,968	0,776	-0,478
	**significance**	0,593	0,614	0,521	0,000	0,516		0,179	0,000	0,000	0,614	0,043	0,000	0,112	0,000	0,305	0,000	0,000	0,000	0,038
**APTT-A**	**correlation coefficient**	0,291	0,036	-0,205	0,724	0,401	0,322	1,000	-0,089	0,075	0,475	-0,120	0,312	-0,435	-0,061	0,656	0,356	0,356	-0,185	0,266
	**significance**	0,227	0,884	0,401	0,000	0,089	0,179		0,718	0,761	0,040	0,624	0,194	0,063	0,803	0,002	0,135	0,135	0,449	0,271
**PCR-A**	**correlation coefficient**	0,090	-0,264	-0,199	0,471	-0,464	0,816	-0,089	1,000	0,939	-0,440	0,719	0,824	0,700	0,832	-0,591	0,789	0,789	0,937	-0,784
	**significance**	0,713	0,274	0,413	0,042	0,045	0,000	0,718		0,000	0,059	0,001	0,000	0,001	0,000	0,008	0,000	0,000	0,000	0,000
**PCT-A**	**correlation coefficient**	0,140	-0,257	-0,241	0,614	-0,377	0,882	0,075	0,939	1,000	-0,326	0,693	0,902	0,608	0,876	-0,466	0,893	0,893	0,871	-0,705
	**significance**	0,569	0,288	0,319	0,005	0,112	0,000	0,761	0,000		0,174	0,001	0,000	0,006	0,000	0,044	0,000	0,000	0,000	0,001
**LEUCO-A**	**correlation coefficient**	-0,561	0,803	0,629	0,147	0,986	-0,124	0,475	-0,440	-0,326	1,000	-0,714	-0,154	-0,888	-0,142	0,354	-0,212	-0,212	-0,436	0,745
	**significance**	0,012	0,000	0,004	0,549	0,000	0,614	0,040	0,059	0,174		0,001	0,530	0,000	0,561	0,137	0,385	0,385	0,062	0,000
**PMN-A**	**correlation coefficient**	0,452	-0,619	-0,572	0,201	-0,752	0,468	-0,120	0,719	0,693	-0,714	1,000	0,494	0,875	0,538	-0,436	0,515	0,515	0,702	-0,956
	**significance**	0,052	0,005	0,011	0,409	0,000	0,043	0,624	0,001	0,001	0,001		0,031	0,000	0,018	0,062	0,024	0,024	0,001	0,000
**Plaq-A**	**correlation coefficient**	0,159	-0,161	-0,198	0,791	-0,196	0,995	0,312	0,824	0,902	-0,154	0,494	1,000	0,403	0,777	-0,255	0,975	0,975	0,775	-0,500
	**significance**	0,516	0,511	0,418	0,000	0,422	0,000	0,194	0,000	0,000	0,530	0,031		0,087	0,000	0,291	0,000	0,000	0,000	0,029
**QuickA**	**correlation coefficient**	0,371	-0,614	-0,489	0,008	-0,874	0,377	-0,435	0,700	0,608	-0,888	0,875	0,403	1,000	0,473	-0,638	0,435	0,435	0,689	-0,939
	**significance**	0,117	0,005	0,034	0,974	0,000	0,112	0,063	0,001	0,006	0,000	0,000	0,087		0,041	0,003	0,063	0,063	0,001	0,000
**DD-A**	**correlation coefficient**	-0,165	-0,007	0,065	0,418	-0,163	0,785	-0,061	0,832	0,876	-0,142	0,538	0,777	0,473	1,000	-0,635	0,733	0,733	0,822	-0,581
	**significance**	0,499	0,977	0,791	0,075	0,504	0,000	0,803	0,000	0,000	0,561	0,018	0,000	0,041		0,003	0,000	0,000	0,000	0,009
**TropUS-A**	**correlation coefficient**	0,343	-0,069	-0,199	0,233	0,298	-0,248	0,656	-0,591	-0,466	0,354	-0,436	-0,255	-0,638	-0,635	1,000	-0,194	-0,194	-0,599	0,605
	**significance**	0,150	0,778	0,413	0,338	0,216	0,305	0,002	0,008	0,044	0,137	0,062	0,291	0,003	0,003		0,426	0,426	0,007	0,006
**FuncProtC**	**correlation coefficient**	0,284	-0,284	-0,317	0,824	-0,259	0,968	0,356	0,789	0,893	-0,212	0,515	0,975	0,435	0,733	-0,194	1,000	1,000	0,717	-0,505
	**significance**	0,239	0,239	0,186	0,000	0,284	0,000	0,135	0,000	0,000	0,385	0,024	0,000	0,063	0,000	0,426		0,000	0,001	0,027
**APACHEII**	**correlation coefficient**	0,284	-0,284	-0,317	0,824	-0,259	0,968	0,356	0,789	0,893	-0,212	0,515	0,975	0,435	0,733	-0,194	1,000	1,000	0,717	-0,505
	**significance**	0,239	0,239	0,186	0,000	0,284	0,000	0,135	0,000	0,000	0,385	0,024	0,000	0,063	0,000	0,426	0,000		0,001	0,027
**PROTOTAL**	**correlation coefficient**	-0,054	-0,160	-0,024	0,344	-0,444	0,776	-0,185	0,937	0,871	-0,436	0,702	0,775	0,689	0,822	-0,599	0,717	0,717	1,000	-0,754
	**significance**	0,827	0,513	0,923	0,150	0,057	0,000	0,449	0,000	0,000	0,062	0,001	0,000	0,001	0,000	0,007	0,001	0,001		0,000
**LAC6H**	**correlation coefficient**	-0,342	0,542	0,473	-0,148	0,758	-0,478	0,266	-0,784	-0,705	0,745	-0,956	-0,500	-0,939	-0,581	0,605	-0,505	-0,505	-0,754	1,000
	**significance**	0,152	0,017	0,041	0,544	0,000	0,038	0,271	0,000	0,001	0,000	0,000	0,029	0,000	0,009	0,006	0,027	0,027	0,000	

Supplementary Table S10. Spearman's correlations in SS non-survivors' group, 2nd sample collected.

		Age	CircHist-B	CitrH3-B	PCA-B	Lactate-B	SOFATot-B	APTT-B	PCR-B	PCT-B	LEUCO-B	PMN-B	Plaq-B	Quick-B	DD-B	TropUS-B	FuncProtC	APACHEII	PROTOTAL	LAC6H
**Age**	**correlation coefficient**	1,000	-0,400	-0,800	0,800	-0,200	0,400	1,000	0,200	0,400	-0,400	0,800	0,600	0,200		0,500	0,600	0,600	-0,500	-0,400
	**significance**		0,385	0,038	0,038	0,717	0,385	0,022	0,717	0,385	0,385	0,038	0,166	0,717		0,422	0,166	0,166	0,422	0,385
**CircHist-B**	**correlation coefficient**	-0,400	1,000	0,800	0,200	0,000	0,400	-0,500	0,000	-0,600	0,600	0,200	0,400	0,000		-1,000	0,400	0,400	1,000	-0,400
	**significance**	0,385		0,038	0,717	1,000	0,385	0,422	1,000	0,166	0,166	0,717	0,385	1,000		0,022	0,385	0,385	0,022	0,385
**CitrH3-B**	**correlation coefficient**	-0,800	0,800	1,000	-0,400	0,400	-0,200	-0,500	-0,400	-0,800	0,800	-0,400	0,000	-0,400		-1,000	0,000	0,000	0,500	0,200
	**significance**	0,038	0,038		0,385	0,385	0,717	0,422	0,385	0,038	0,038	0,385	1,000	0,385		0,022	1,000	1,000	0,422	0,717
**PCA-B**	**correlation coefficient**	0,800	0,200	-0,400	1,000	-0,400	0,800	0,500	0,400	0,200	-0,200	1,000	0,800	0,400		-0,500	0,800	0,800	0,500	-0,800
	**significance**	0,038	0,717	0,385		0,385	0,038	0,422	0,385	0,717	0,717	0,001	0,038	0,385		0,422	0,038	0,038	0,422	0,038
**Lactate-B**	**correlation coefficient**	-0,200	0,000	0,400	-0,400	1,000	-0,800	1,000	-1,000	-0,800	0,800	-0,400	0,200	-1,000		0,500	0,200	0,200	-0,500	0,800
	**significance**	0,717	1,000	0,385	0,385		0,038	0,022	0,001	0,038	0,038	0,385	0,717	0,001		0,422	0,717	0,717	0,422	0,038
**SOFATot-B**	**correlation coefficient**	0,400	0,400	-0,200	0,800	-0,800	1,000	-0,500	0,800	0,400	-0,400	0,800	0,400	0,800		-1,000	0,400	0,400	0,500	-1,000
	**significance**	0,385	0,385	0,717	0,038	0,038		0,422	0,038	0,385	0,385	0,038	0,385	0,038		0,022	0,385	0,385	0,422	0,001
**APTT-B**	**correlation coefficient**	1,000	-0,500	-0,500	0,500	1,000	-0,500	1,000	-1,000	-0,500	0,500	0,500	0,500	-1,000		0,500	0,500	0,500	-1,000	0,500
	**significance**	0,022	0,422	0,422	0,422	0,022	0,422		0,022	0,422	0,422	0,422	0,422	0,022		0,422	0,422	0,422	0,333	0,422
**PCR-B**	**correlation coefficient**	0,200	0,000	-0,400	0,400	-1,000	0,800	-1,000	1,000	0,800	-0,800	0,400	-0,200	1,000		-0,500	-0,200	-0,200	0,500	-0,800
	**significance**																			
**PCT-B**	**correlation coefficient**	0,400	-0,600	-0,800	0,200	-0,800	0,400	-0,500	0,800	1,000	-1,000	0,200	-0,400	0,800		0,500	-0,400	-0,400	-0,500	-0,400
	**significance**	0,717	1,000	0,385	0,385	0,001	0,038	0,022		0,038	0,038	0,385	0,717	0,001		0,422	0,717	0,717	0,422	0,038
**LEUCO-B**	**correlation coefficient**	-0,400	0,600	0,800	-0,200	0,800	-0,400	0,500	-0,800	-1,000	1,000	-0,200	0,400	-0,800		-0,500	0,400	0,400	0,500	0,400
	**significance**	0,385	0,166	0,038	0,717	0,038	0,385	0,422	0,038	0,001		0,717	0,385	0,038		0,422	0,385	0,385	0,422	0,385
**PMN-B**	**correlation coefficient**	0,800	0,200	-0,400	1,000	-0,400	0,800	0,500	0,400	0,200	-0,200	1,000	0,800	0,400		-0,500	0,800	0,800	0,500	-0,800
	**significance**	0,038	0,717	0,385	0,001	0,385	0,038	0,422	0,385	0,717	0,717		0,038	0,385		0,422	0,038	0,038	0,422	0,038
**Plaq-B**	**correlation coefficient**	0,600	0,400	0,000	0,800	0,200	0,400	0,500	-0,200	-0,400	0,400	0,800	1,000	-0,200		-0,500	1,000	1,000	0,500	-0,400
	**significance**	0,166	0,385	1,000	0,038	0,717	0,385	0,422	0,717	0,385	0,385	0,038		0,717		0,422	0,001	0,001	0,422	0,385
**Quick-B**	**correlation coefficient**	0,200	0,000	-0,400	0,400	-1,000	0,800	-1,000	1,000	0,800	-0,800	0,400	-0,200	1,000		-0,500	-0,200	-0,200	0,500	-0,800
	**significance**	0,717	1,000	0,385	0,385	0,001	0,038	0,022	0,001	0,038	0,038	0,385	0,717			0,422	0,717	0,717	0,422	0,038
**DD-B**	**correlation coefficient**																			
	**significance**																			
**TropUS-B**	**correlation coefficient**	0,500	-1,000	-1,000	-0,500	0,500	-1,000	0,500	-0,500	0,500	-0,500	-0,500	-0,500	-0,500		1,000	-0,500	-0,500	-1,000	1,000
	**significance**	0,422	0,022	0,022	0,422	0,422	0,022	0,422	0,422	0,422	0,422	0,422	0,422	0,422			0,422	0,422	0,333	0,022
**FuncProtC**	**correlation coefficient**	0,600	0,400	0,000	0,800	0,200	0,400	0,500	-0,200	-0,400	0,400	0,800	1,000	-0,200		-0,500	1,000	1,000	0,500	-0,400
	**significance**	0,166	0,385	1,000	0,038	0,717	0,385	0,422	0,717	0,385	0,385	0,038	0,001	0,717		0,422		0,001	0,422	0,385
**APACHEII**	**correlation coefficient**	0,600	0,400	0,000	0,800	0,200	0,400	0,500	-0,200	-0,400	0,400	0,800	1,000	-0,200		-0,500	1,000	1,000	0,500	-0,400
	**significance**	0,166	0,385	1,000	0,038	0,717	0,385	0,422	0,717	0,385	0,385	0,038	0,001	0,717		0,422	0,001		0,422	0,385
**PROTOTAL**	**correlation coefficient**	-0,500	1,000	0,500	0,500	-0,500	0,500	-1,000	0,500	-0,500	0,500	0,500	0,500	0,500		-1,000	0,500	0,500	1,000	-0,500
	**significance**	0,422	0,022	0,422	0,422	0,422	0,422	0,333	0,422	0,422	0,422	0,422	0,422	0,422		0,333	0,422	0,422		0,422
**LAC6H**	**correlation coefficient**	-0,400	-0,400	0,200	-0,800	0,800	-1,000	0,500	-0,800	-0,400	0,400	-0,800	-0,400	-0,800		1,000	-0,400	-0,400	-0,500	1,000
	**significance**	0,385	0,385	0,717	0,038	0,038	0,001	0,422	0,038	0,385	0,385	0,038	0,385	0,038		0,022	0,385	0,385	0,422	

Supplementary Table S11. Spearman's correlations in SS non-survivors' group, 3rd sample collected.

		Age	CircHist-C	CitrH3-C	PCA-C	Lactate-C	SOFATot-C	APTT-C	PCR-C	PCT-C	LEUCO-C	PMN-C	Plaq-C	Quick-C	DD-C	TropUS-C	FuncProtC	APACHEII	PROTOTAL	LAC6H
**Age**	**correlation coefficient**	1,000	0,800	-0,200	0,400	-0,400	0,400	1,000	-0,600	0,400	0,800	-0,200	1,000	0,400		0,500	0,600	0,600	-0,500	-0,400
	**significance**		0,038	0,717	0,385	0,385	0,385	0,022	0,166	0,385	0,038	0,717	0,001	0,385		0,422	0,166	0,166	0,422	0,385
**CircHist-C**	**correlation coefficient**	0,800	1,000	0,400	-0,200	0,000	0,000	1,000	-0,800	-0,200	1,000	-0,400	0,800	-0,200		0,500	0,800	0,800	-0,500	0,000
	**significance**	0,038		0,385	0,717	1,000	1,000	0,022	0,038	0,717	0,001	0,385	0,038	0,717		0,422	0,038	0,038	0,422	1,000
**CitrH3-C**	**correlation coefficient**	-0,200	0,400	1,000	-0,800	0,800	-0,800	1,000	-0,200	-0,800	0,400	-0,600	-0,200	-0,800		0,500	0,200	0,200	-0,500	0,800
	**significance**	0,717	0,385		0,038	0,038	0,038	0,022	0,717	0,038	0,385	0,166	0,717	0,038		0,422	0,717	0,717	0,422	0,038
**PCA-C**	**correlation coefficient**	0,400	-0,200	-0,800	1,000	-0,400	0,400	-0,500	0,400	1,000	-0,200	0,000	0,400	1,000		0,500	-0,400	-0,400	-0,500	-0,400
	**significance**	0,385	0,717	0,038		0,385	0,385	0,422	0,385	0,001	0,717	1,000	0,385	0,001		0,422	0,385	0,385	0,422	0,385
**Lactate-C**	**correlation coefficient**	-0,400	0,000	0,800	-0,400	1,000	-1,000	0,500	0,400	-0,400	0,000	-0,800	-0,400	-0,400		1,000	-0,400	-0,400	-0,500	1,000
	**significance**	0,385	1,000	0,038	0,385		0,001	0,422	0,385	0,385	1,000	0,038	0,385	0,385		0,022	0,385	0,385	0,422	0,001
**SOFATot-C**	**correlation coefficient**	0,400	0,000	-0,800	0,400	-1,000	1,000	-0,500	-0,400	0,400	0,000	0,800	0,400	0,400		-1,000	0,400	0,400	0,500	-1,000
	**significance**	0,385	1,000	0,038	0,385	0,001		0,422	0,385	0,385	1,000	0,038	0,385	0,385		0,022	0,385	0,385	0,422	0,001
**APTT-C**	**correlation coefficient**	1,000	1,000	1,000	-0,500	0,500	-0,500	1,000	-0,500	-0,500	1,000	-0,500	1,000	-0,500		0,500	0,500	0,500	-1,000	0,500
	**significance**	0,022	0,022	0,022	0,422	0,422	0,422		0,422	0,422	0,022	0,422	0,022	0,422		0,422	0,422	0,422	0,333	0,422
**PCR-C**	**correlation coefficient**	-0,600	-0,800	-0,200	0,400	0,400	-0,400	-0,500	1,000	0,400	-0,800	-0,200	-0,600	0,400		0,500	-1,000	-1,000	-0,500	0,400
	**significance**	0,166	0,038	0,717	0,385	0,385	0,385	0,422		0,385	0,038	0,717	0,166	0,385		0,422	0,001	0,001	0,422	0,385
**PCT-C**	**correlation coefficient**	0,400	-0,200	-0,800	1,000	-0,400	0,400	-0,500	0,400	1,000	-0,200	0,000	0,400	1,000		0,500	-0,400	-0,400	-0,500	-0,400
	**significance**	0,385	0,717	0,038	0,001	0,385	0,385	0,422	0,385		0,717	1,000	0,385	0,001		0,422	0,385	0,385	0,422	0,385
**LEUCO-C**	**correlation coefficient**	0,800	1,000	0,400	-0,200	0,000	0,000	1,000	-0,800	-0,200	1,000	-0,400	0,800	-0,200		0,500	0,800	0,800	-0,500	0,000
	**significance**	0,038	0,001	0,385	0,717	1,000	1,000	0,022	0,038	0,717		0,385	0,038	0,717		0,422	0,038	0,038	0,422	1,000
**PMN-C**	**correlation coefficient**	-0,200	-0,400	-0,600	0,000	-0,800	0,800	-0,500	-0,200	0,000	-0,400	1,000	-0,200	0,000		-1,000	0,200	0,200	1,000	-0,800
	**significance**	0,717	0,385	0,166	1,000	0,038	0,038	0,422	0,717	1,000	0,385		0,717	1,000		0,022	0,717	0,717	0,022	0,038
**Plaq-C**	**correlation coefficient**	1,000	0,800	-0,200	0,400	-0,400	0,400	1,000	-0,600	0,400	0,800	-0,200	1,000	0,400		0,500	0,600	0,600	-0,500	-0,400
	**significance**	0,001	0,038	0,717	0,385	0,385	0,385	0,022	0,166	0,385	0,038	0,717		0,385		0,422	0,166	0,166	0,422	0,385
**Quick-C**	**correlation coefficient**	0,400	-0,200	-0,800	1,000	-0,400	0,400	-0,500	0,400	1,000	-0,200	0,000	0,400	1,000		0,500	-0,400	-0,400	-0,500	-0,400
	**significance**	0,385	0,717	0,038	0,001	0,385	0,385	0,422	0,385	0,001	0,717	1,000	0,385			0,422	0,385	0,385	0,422	0,385
**DD-C**	**correlation coefficient**																			
	**significance**																			
**TropUS-C**	**correlation coefficient**	0,500	0,500	0,500	0,500	1,000	-1,000	0,500	0,500	0,500	0,500	-1,000	0,500	0,500		1,000	-0,500	-0,500	-1,000	1,000
	**significance**	0,422	0,422	0,422	0,422	0,022	0,022	0,422	0,422	0,422	0,422	0,022	0,422	0,422			0,422	0,422	0,333	0,022
**FuncProtC**	**correlation coefficient**	0,600	0,800	0,200	-0,400	-0,400	0,400	0,500	-1,000	-0,400	0,800	0,200	0,600	-0,400		-0,500	1,000	1,000	0,500	-0,400
	**significance**	0,166	0,038	0,717	0,385	0,385	0,385	0,422	0,001	0,385	0,038	0,717	0,166	0,385		0,422		0,001	0,422	0,385
**APACHEII**	**correlation coefficient**	0,600	0,800	0,200	-0,400	-0,400	0,400	0,500	-1,000	-0,400	0,800	0,200	0,600	-0,400		-0,500	1,000	1,000	0,500	-0,400
	**significance**	0,166	0,038	0,717	0,385	0,385	0,385	0,422	0,001	0,385	0,038	0,717	0,166	0,385		0,422	0,001		0,422	0,385
**PROTOTAL**	**correlation coefficient**	-0,500	-0,500	-0,500	-0,500	-0,500	0,500	-1,000	-0,500	-0,500	-0,500	1,000	-0,500	-0,500		-1,000	0,500	0,500	1,000	-0,500
	**significance**	0,422	0,422	0,422	0,422	0,422	0,422	0,333	0,422	0,422	0,422	0,022	0,422	0,422		0,333	0,422	0,422		0,422
**LAC6H**	**correlation coefficient**	-0,400	0,000	0,800	-0,400	1,000	-1,000	0,500	0,400	-0,400	0,000	-0,800	-0,400	-0,400		1,000	-0,400	-0,400	-0,500	1,000
	**significance**	0,385	1,000	0,038	0,385	0,001	0,001	0,422	0,385	0,385	1,000	0,038	0,385	0,385		0,022	0,385	0,385	0,422	
